# Multi-Omics profiling identifies aldehyde dehydrogenase 2 as a critical mediator in the crosstalk between Treg-mediated immunosuppression microenvironment and hepatocellular carcinoma

**DOI:** 10.7150/ijbs.93075

**Published:** 2024-05-05

**Authors:** Zhi-Yong Liu, Xia-Hui Lin, Hong-Ying Guo, Xuan Shi, Dan-Ying Zhang, Jia-Lei Sun, Guang-Cong Zhang, Ru-Chen Xu, Fu Wang, Xiang-Nan Yu, Dou Wang, Shu-Qiang Weng, Xi-Zhong Shen, Tao-Tao Liu, Ling Dong, Ji-Min Zhu

**Affiliations:** 1 Department of Gastroenterology and Hepatology, Zhongshan Hospital of Fudan University, Shanghai 200030, China.; 2 Shanghai Institute of Liver Diseases, Shanghai 200030, China.; 3 Department of Gastroenterology, Shigatse People's Hospital, Shigatse, Tibet 857000, China.; 4 Key Laboratory of Medical Molecular Virology, Shanghai Medical College of Fudan University, Shanghai 200030, China.

**Keywords:** ALDH2, Metabolic disturbance, TNFRSF18 (GITR), Regulatory T cells, Liver cancer

## Abstract

Dysregulation of the aldehyde dehydrogenase (ALDH) family has been implicated in various pathological conditions, including cancer. However, a systematic evaluation of ALDH alterations and their therapeutic relevance in hepatocellular carcinoma (HCC) remains lacking. Herein, we found that 15 of 19 ALDHs were transcriptionally dysregulated in HCC tissues compared to normal liver tissues. A four gene signature, including ALDH2, ALDH5A1, ALDH6A1, and ALDH8A1, robustly predicted prognosis and defined a high-risk subgroup exhibiting immunosuppressive features like regulatory T cell (Tregs) infiltration. Single-cell profiling revealed selective overexpression of tumor necrosis factor receptor superfamily member 18 (TNFRSF18) on Tregs, upregulated in high-risk HCC patients. We identified ALDH2 as a tumor suppressor in HCC, with three novel phosphorylation sites mediated by protein kinase C zeta that enhanced enzymatic activity. Mechanistically, ALDH2 suppressed Tregs differentiation by inhibiting β-catenin/TGF-β1 signaling in HCC. Collectively, our integrated multi-omics analysis defines an ALDH-Tregs-TNFRSF18 axis that contributes to HCC pathogenesis and represents potential therapeutic targets for this aggressive malignancy.

## Introduction

Hepatocellular carcinoma (HCC) comprises one of the most common pathological entities of primary liver cancer, with high morbidity and mortality rates worldwide 1. While surgery, liver transplantation, and chemotherapy remain the primary HCC treatments 2, 5-year survival rates continue to be unsatisfactory due to early recurrence, distant metastasis, and therapy resistance 3. Additionally, as most HCC patients are diagnosed at later stages, treatment options become limited, resulting in poor prognosis. Recently, aberrant metabolic reprogramming has been identified as an emerging hallmark of cancer 4, 5. Given the liver's critical metabolic functions, mounting evidence indicates that metabolic dysregulations are implicated in HCC initiation and progression 6-8. Thus, targeting metabolic changes may serve as a promising therapeutic strategy for HCC.

The aldehyde dehydrogenase (ALDH) family of 19 enzymes localized in cellular compartments, such as mitochondria, catalyze aldehydes to carboxylic acids 9, 10. ALDH dysregulation causes aberrant carbonyl metabolism implicated in cancers 11. Inhibiting ALDH1A accumulates intracellular toxic aldehydes, inducing DNA damage in ovarian cancer 12. Up-regulated ALDH1A1 is acknowledged as a cancer stem cell marker 13, 14. RNA sequencing revealed that ALDH expression associates with HCC prognosis, nominating the family as biomarkers and therapeutic targets 15. Also, ALDH2 polymorphism and alcoholics were identified as two risk factors for HCC development. ALDH2 deficiency specifically promotes alcohol-related HCC progression from fibrosis by shuttling oxidized mitochondrial DNA into neighboring cells, activating the oncogenic signaling 16. Furthermore, ALDH7A1 also enhances energy homeostasis in HCC cells under nutrient deprivation 17. While ALDHs are clearly implicated in HCC, a comprehensive characterization of the family is lacking. Investigating coordinated ALDH expression patterns could uncover key members driving pathogenesis, providing approaches to target this metabolic pathway.

Within the tumor microenvironment (TME), regulatory T cells (Tregs) are a subset of CD4^+^ T cells that mediate immune tolerance. Tregs can be classified into two main types based on their origin: thymic Tregs (tTregs) and peripheral Tregs (pTregs). tTregs are tissue-intrinsic and are produced in the thymus, while pTregs differentiate from conventional T cells in peripheral tissues or are induced by TGF-β 18. In many tumor tissues, Tregs play immunosuppressive functions and can be identified by the expression of specific surface markers such as IL2RA (interleukin 2 receptor alpha, also called CD25) and FOXP3, which is associated with poor prognosis 19. Tumor necrosis factor receptor superfamily member 18 (TNFRSF18), also known as glucocorticoid-induced tumor necrosis factor receptor-related protein (GITR), is expressed on a small population of CD4^+^ and CD8^+^ T cells 20. Importantly, several studies have found that TNFRSF18 is a marker of Tregs and is associated with Treg activation in both human and murine models 20, 21, suggesting its potential as a target for anti-tumor immunotherapy.

Herein, this study conducted a comprehensive multi-omics interrogation of the 19 ALDH family members in HCC. Transcriptional profiles, genetic variants, protein expression, and post-translational regulation across publicly available HCC datasets were analyzed. A 4-ALDH gene signature robustly predicted prognosis. Single-cell RNA-sequencing revealed ALDH dysregulation associates with a suppressive tumor microenvironment, marked by Tregs infiltration and TNFRSF18 upregulation. Mechanistic experiments identified ALDH2 as a key HCC tumor suppressor, finding its overexpression attenuated Treg by suppressing the β-catenin and TGF-β1 signaling.

## Materials and Methods

### Retrieval of raw data for analysis

The RNA sequencing data of transcripts per million (TPM) and clinical features of patients were obtained from the LIHC (liver hepatocellular carcinoma) project of TCGA (https://portal.gdc.cancer.gov/) for the training set. The RNA sequencing data, proteome data, phosphoproteome data, and clinicopathological information of 159 HCC patients in Zhongshan Hospital, Fudan University, HCC research (ZS-HCC) were downloaded from a dataset of OEP000321 18 in the NODE (National Omics Data Encyclopedia) (https://www.biosino.org/node) for the validation set, due to the integrated multi-omics HCC data (**Supplementary Table 3**). Single-cell sequencing data of 6 HCC patients was obtained from dataset CNP0000650 19 in CNGBdb (China National Gene Bank Data Base) (https://db.cngb.org/). Coy number variation (CNV) data of the ALDH family were obtained from cBioPortal (http://www.cbioportal.org/) 20.

### Cell lines

The human HCC cell line (PLC/PRF/5) and mouse HCC cell line (Hepa1-6) were purchased from the Cell Bank of the Chinese Academy of Sciences (Shanghai, China) and cultured in Dulbecco's modified Eagle's medium (KeyGEN, Nanjing, China) containing 10% fetal bovine serum (FBS) (Sigma, Saint Louis, USA) and 100 U/mL of penicillin and 50 μg/mL streptomycin (Gibco, California, USA) in a 37 °C humidified incubator with 5% CO_2_.

### Clinical samples

The clinical tumor and para-tumor tissues of HCC were obtained from 30 patients from Zhongshan Hospital, Fudan University, with informed patient consent. Fresh tissues were harvested and stored in liquid nitrogen for further application. This study was approved by the Institutional Research Ethics Committee of Zhongshan Hospital.

### Animal study

Male C57BL/6N mice aged 5 weeks were obtained from Charles River Laboratories. All mice were maintained under specific pathogen-free conditions. The animal study was approved by the Animal Care and Use Committee at Zhongshan Hospital of Fudan University. 5 × 10^6^ LV-ALDH2 and LV-control Hepa1-6 cells were subcutaneously injected into the back flanks of two C57BL/6N mice. After 2 weeks, the subcutaneous tumors were resected into 3 mm^3^ tissue masses and planted into mice livers to establish an orthotopic xenograft model. The xenograft mice were examined using magnetic resonance imaging (MRI) and sacrificed after 3 weeks. The tumor volumes were recorded and calculated using the formula length × width^2^ × 0.5.

### Flow cytometry (FCM)

Tumor tissues of mice were cut into small pieces and lysed using collagenase IV (1 mg/mL, Sigma, USA) and DNase I (Invitrogen, USA) for 1 h at 37 °C. Then, we filtrated the tissue medium using a 70 μm filter to obtain single-cell suspensions. The cell suspensions were stained with antibodies for 30 minutes on ice and subjected to FCM analysis. The following reagents and antibodies were used: LIVE/DEAD™ Fixable Stain (Invitrogen, California, USA), anti-mouse CD45-BV510 (Biolegend, California, USA), anti-mouse CD3-BUV395 (BD, New Jersey, USA), anti-mouse CD4-FITC (BD, New Jersey, USA), anti-mouse CD8-PerCP-Cy5.5 (BD, New Jersey, USA), anti-mouse FOXP3-PE (Biolegend, California, USA), anti-mouse CD25-PE-cy7 (Biolegend, California, USA), anti-mouse CD127-BV711 (BD, New Jersey, USA ).

### Statistical analysis

All data were expressed as mean ± standard deviation (SD). The statistical significance of categorical variables was analyzed by the Chi-square test or Fisher's exact test. For continuous variables, the student's t-test or one-way ANOVA was applied to analyze the differences. The correlation coefficients between two variables were explored using Pearson correlation analysis. The Kaplan-Meier analysis was used to compare overall survival (OS) and disease-free survival (DFS) proportions between the two groups. Univariate and multivariate Cox regression analyses with hazard ratios (HRs) and 95% confidence intervals (CIs) were applied to examine the independent prognostic factors. All statistical analyses were performed using R studio and GraphPad Prism 9. *p* < 0.05 were considered statistically significant.

## Results

### Expression of the ALDH family members in HCC

**Figure 1:**
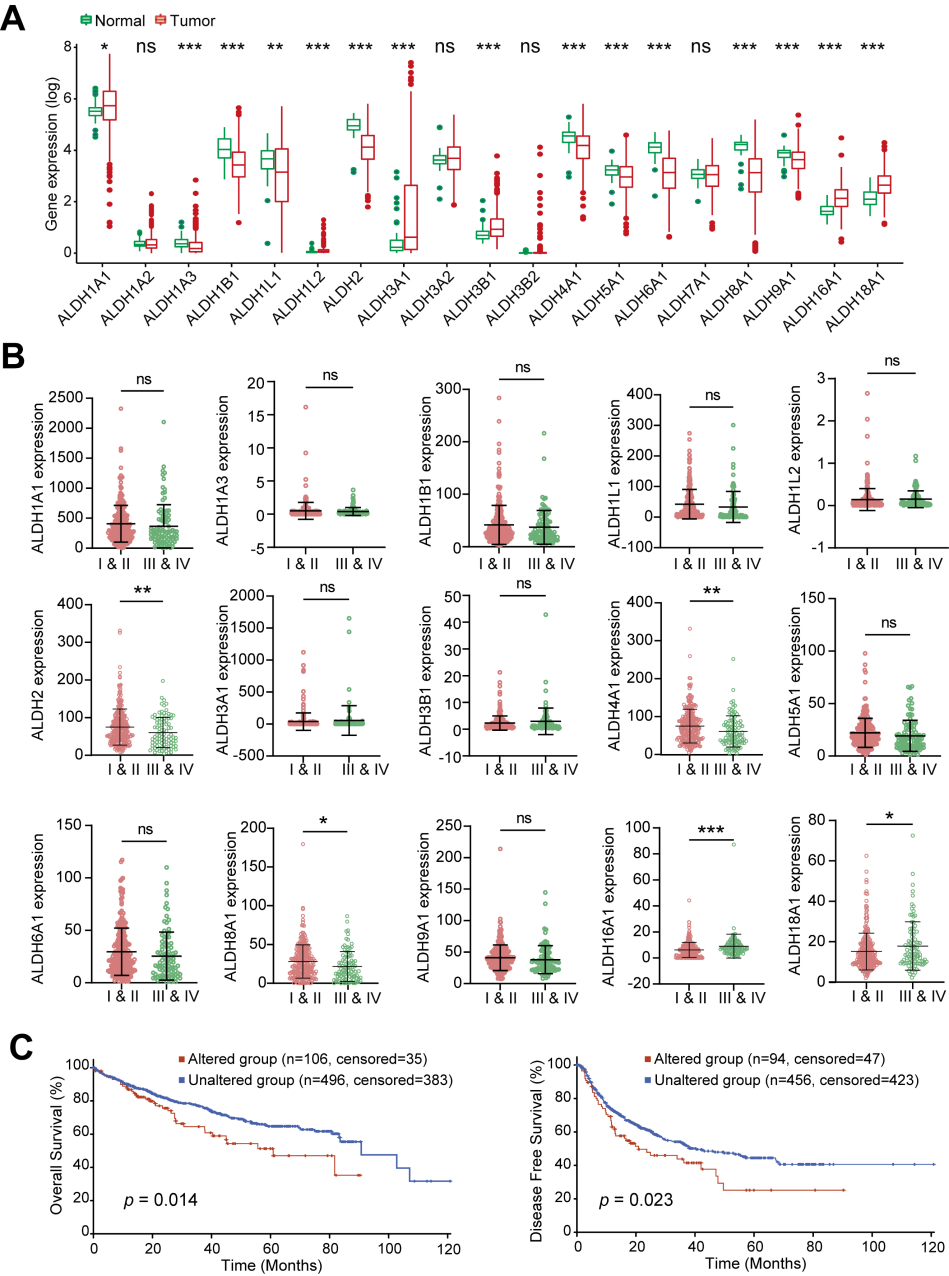
** Dysregulated Expression and Clinical Impact of ALDHs in HCC.** (A) Differential expression analysis of 19 ALDH genes in 379 HCC tumors versus 59 normal liver tissues from TCGA. Boxplots depict 6 upregulated (ALDH1A1, ALDH1L2, ALDH3A1, ALDH3B1, ALDH16A1, ALDH18A1) and 9 downregulated (ALDH1A3, ALDH1B1, ALDH1L1, ALDH2, ALDH4A1, ALDH5A1, ALDH6A1, ALDH8A1, ALDH9A1) ALDHs in HCC. (B) ALDH2, ALDH4A1, ALDH8A1 exhibited lower expression while ALDH16A1, ALDH18A1 showed higher levels in advanced stage III/IV HCC patients compared to early stages I/II. (C) Kaplan-Meier analysis demonstrating significantly reduced overall survival (left) and disease-free survival (right) in HCC patients harboring mutations in ALDH genes. Unpaired Student's t-test in (A, B); Log-rank test in (C). * *p* < 0.05, ** *p* < 0.01, *** *p* < 0.001.

Transcriptomic profiling across 379 HCC tumors and 59 normal liver tissues from TCGA-LIHC dataset revealed widespread dysregulation of the ALDH family in HCC. Of the 19 ALDH genes analyzed, 6 (ALDH1A1, ALDH1L2, ALDH3A1, ALDH3B1, ALDH16A1, ALDH18A1) were upregulated, while 9 (ALDH1A3, ALDH1B1, ALDH1L1, ALDH2, ALDH4A1, ALDH5A1, ALDH6A1, ALDH8A1, ALDH9A1) showed downregulation in tumors compared to normal tissue (**Figure 1A**). When stratifying ALDH expression across clinical stages, advanced-stage (III and IV) patients exhibited significantly lower levels of ALDH2, ALDH4A1, ALDH8A1 and higher ALDH16A1, ALDH18A1 compared to early-stage (I and II), while the remaining 10 ALDHs showed no stage-associated differences (**Figure 1B**). Pearson correlation revealed complex co-expression patterns among ALDH family members in HCC (**Figure S1A**).

To explore genetic mechanisms underlying the observed transcriptional changes, we searched for copy number variations (CNVs) and DNA mutations affecting ALDH genes using the cBioPortal tool. No evident CNV clusters were identified across chromosomes (**Figure S1B**). Targeted mutational analysis revealed relatively infrequent but clinically impactful ALDH alterations in HCC patients (**Figure S1C**). Specifically, HCC cases harboring ALDH mutations exhibited significantly worse overall survival (OS; *p* = 0.014) and disease-free survival (DFS; *p* = 0.023) compared to wild-type cases (**Figure 1C**). Collectively, these multi-omics data highlight widespread but coordinated expression changes, genetic underpinnings, and prognostic associations of the ALDH enzyme family in HCC.

### Prognostic utility of an ALDH gene signature in HCC

**Figure 2:**
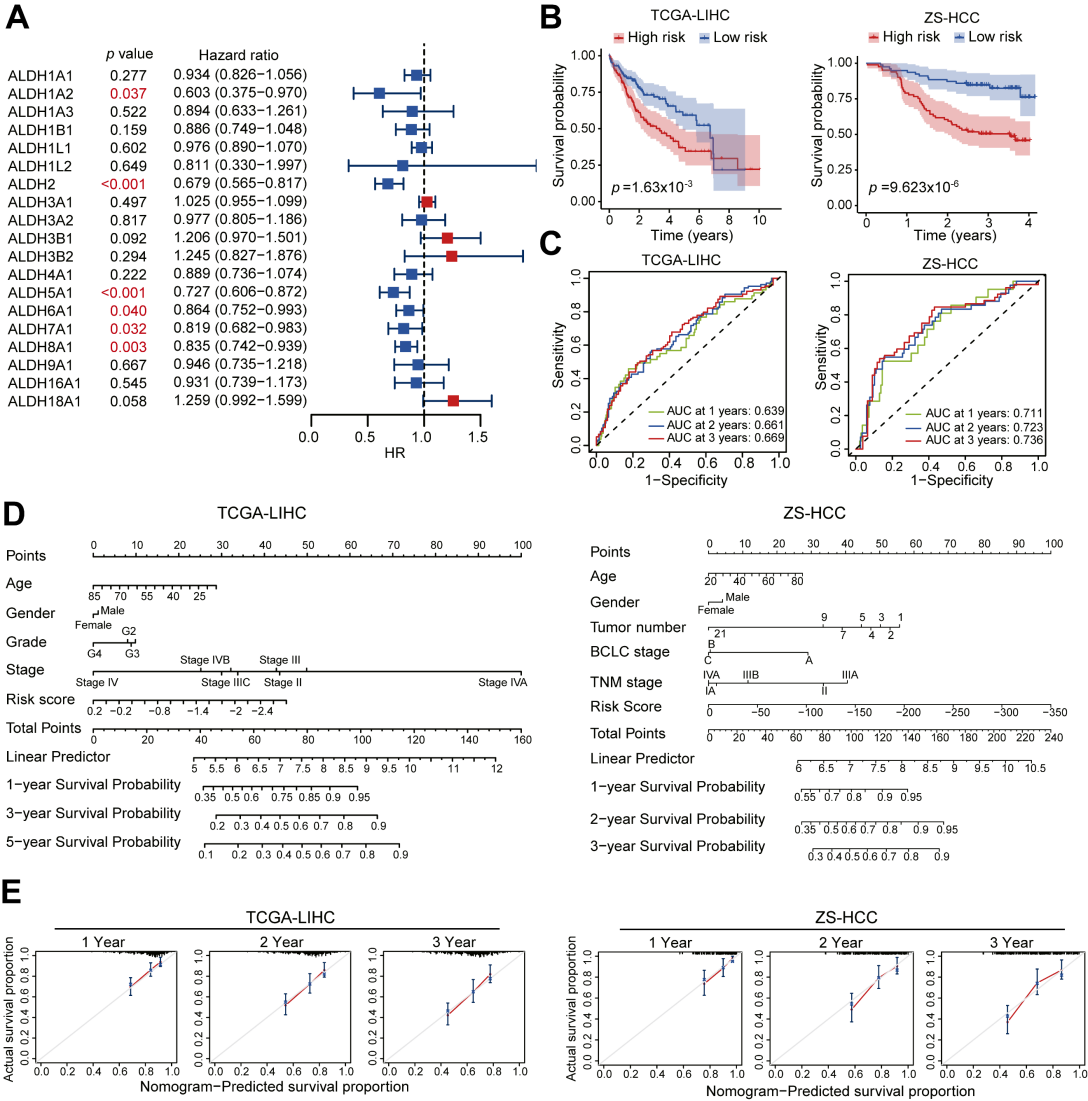
** An ALDH gene expression signature defines a robust prognostic risk model in HCC.** (A) Univariate Cox regression analysis of ALDH gene expression and overall survival in TCGA-LIHC cohort. Six genes (ALDH1A2, ALDH2, ALDH5A1, ALDH6A1, ALDH7A1, ALDH8A1) were significantly associated with prognosis (red). (B) Kaplan-Meier survival curves stratified by high- vs. low-risk groups based on a 4-gene signature (ALDH2, ALDH5A1, ALDH6A1, ALDH8A1) in TCGA-LIHC training set (n = 361, left) and an independent ZS-HCC validation cohort (n= 159, right). High-risk patients exhibited significantly reduced overall survival. (C) Time-dependent receiver operating characteristic (ROC) curves demonstrate robust prognostic performance of the 4-ALDH risk model in TCGA-LIHC (left) and ZS-HCC (right) cohorts across 1-, 2-, and 3-year overall survival. (D) A nomogram integrating the ALDH risk score with clinicopathologic features to facilitate individualized survival prediction in HCC patients across TCGA-LIHC and ZS-HCC cohorts. (E) Calibration plots confirm excellent agreement between predicted and observed 1-, 2-, and 3-year overall survival probabilities using the nomogram in both TCGA-LIHC (left) and ZS-HCC (right) datasets.

To comprehensively assess the prognostic associations of ALDH expression in HCC, a univariate Cox regression analysis was conducted across the 19 ALDH genes using TCGA data. Six candidates (ALDH1A2, ALDH2, ALDH5A1, ALDH6A1, ALDH7A1, ALDH8A1) emerged as significantly associated with overall survival (*p* < 0.05; **Figure 2A**). Based on the Cox regression results and their differential expression patterns, we focused on a four-gene signature comprising ALDH2, ALDH5A1, ALDH6A1, and ALDH8A1 as robust prognostic predictors. Using LASSO regression analysis, we calculated risk scores for each HCC patient in TCGA-LIHC training and an independent validation (ZS-HCC) cohort by integrating this ALDH signature with their expression values (**Figure S2A-S2B**).

Stratifying patients into high- versus low-risk groups based on median risk score revealed significantly shorter overall survival among high-risk cases in both the training (*p* = 1.63×10^-3^) and validation (*p* = 9.62×10^-6^) sets (**Figure 2B**). Time-dependent receiver operating characteristic (ROC) curve analysis further confirmed the robust prognostic performance of this ALDH risk model, with AUCs of 0.639-0.669 (TCGA) and 0.711-0.736 (ZS-HCC) for 1-, 2-, and 3-year OS (**Figure 2C**).

A nomogram integrating the ALDH risk score with clinicopathologic features (such as age, gender, and tumor stage) facilitated individualized survival prediction (**Figure 2D**), showing excellent calibration between the predicated 1-, 2-, and 3-year survival rates and the actual prognosis outcomes of the HCC patients across both cohorts (**Figure 2E**). Multivariate Cox analysis identified that tumor stage (*p* < 0.001, HR = 2.26, 95% CI, 1.57-3.24) and risk score (*p* < 0.001, HR = 2.38, 95% CI, 1.51-3.77) are two significant prognostic factors (**Table 1**). Patients stratified by low expression of the four-gene signature consistently associated with poor outcomes across both cohorts by Kaplan-Meier curves (**Figure S2C-S2D**). Together, this integrated multi-cohort analysis established a 4-ALDH gene signature as a powerful and clinically applicable prognostic classifier in HCC. The robust risk model enables prediction of overall survival, highlighting metabolic vulnerabilities as potential therapeutic targets.

### Immunosuppressive tumor microenvironment associated with high ALDH risk score

**Figure 3:**
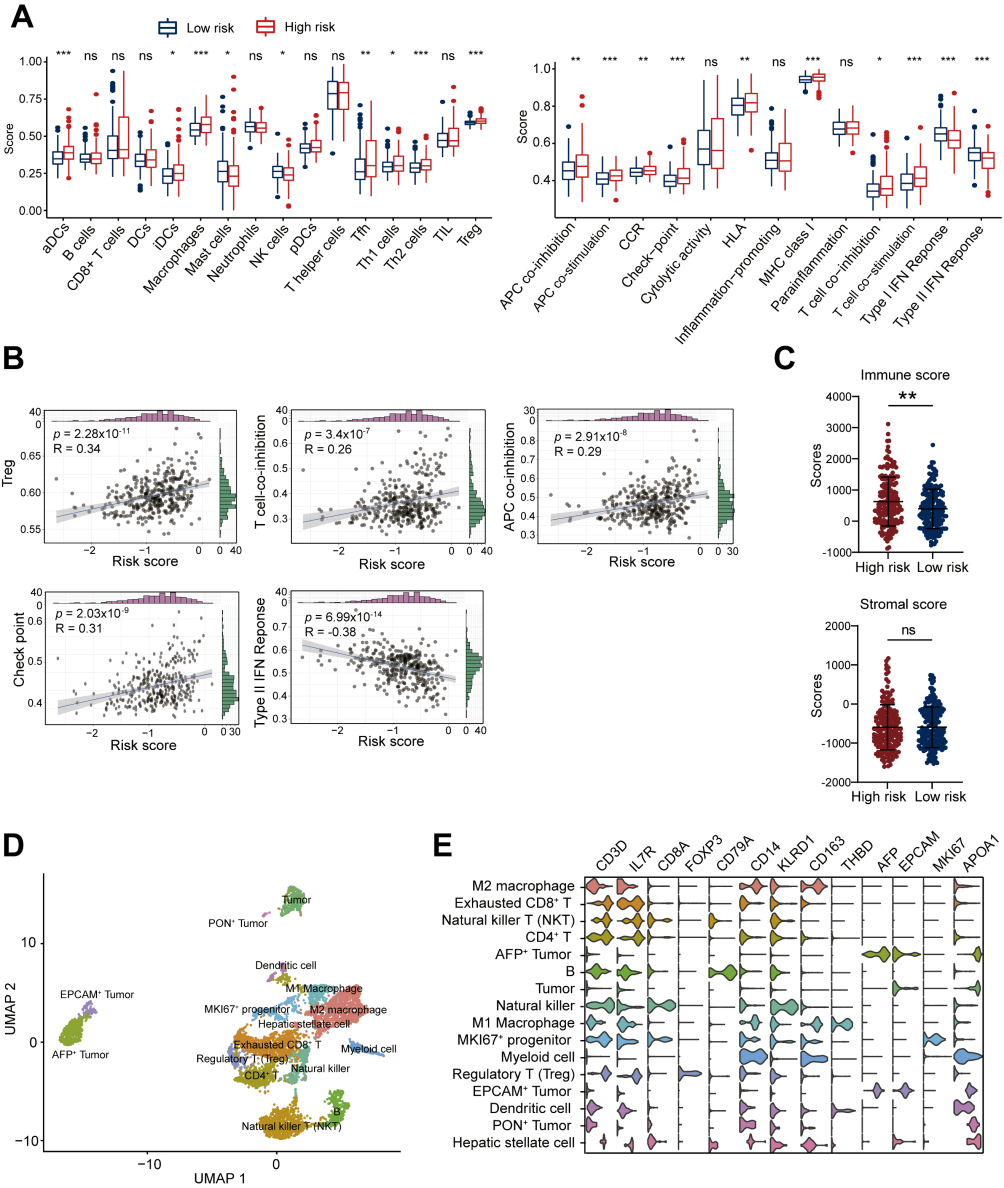
** High ALDH risk score defines an immunosuppressive tumor microenvironment in HCC.** (A) Single-sample gene set enrichment analysis (ssGSEA) comparing immune cell infiltration and functions between high- and low-risk ALDH groups in the TCGA-LIHC cohort. High-risk tumors exhibited increased macrophages, Tregs, and suppression of anti-tumor immunity. (B) The ALDH risk score positively correlated with Treg infiltration, T cell co-inhibition, antigen-presenting cell (APC) co-inhibition, and checkpoint expression, while negatively associating with type II interferon response in HCC. (C) Immune cytolytic scoring revealed elevated immune infiltration but comparable stromal content in the high- versus low-risk ALDH groups. (D) Uniform Manifold Approximation and Projection (UMAP) of 19,126 single-cell transcriptomes from 6 HCC patients, defining 16 major cell populations in the tumor microenvironment. (E) Violin plots depicting expression of canonical marker genes across the 16 clusters, highlighting the presence of exhausted CD8^+^ T cells, immunosuppressive M2 macrophages, and Tregs. Unpaired student's *t*-test was used in (A, C). Pearson correlation analysis was used in (B). * *p* < 0.05, ** *p* < 0.01, *** *p* < 0.001, ns, not significant.

Given the critical role of the tumor immune microenvironment in cancer progression, we investigated associations between the ALDH risk model and immune cell infiltration/function using ssGSEA R package of TCGA-LIHC dataset. Comparing high- versus low-risk HCC groups revealed elevated infiltration of immunosuppressive cell types like macrophages and Tregs in the high-risk cohort (**Figure 3A**). Moreover, the ALDH risk score was positively related to Treg levels (R = 0.34, *p* = 2.28×10^-11^, *n* = 361), T cell co-inhibition (R = 0.26, *p* = 3.4×10^-7^, *n* = 361), antigen-presenting cell co-inhibition (R = 0.29, *p* = 2.91×10^-8^, *n* = 361), and checkpoint expression (R = 0.31, *p* = 2.03×10^-9^, *n* = 361); while it was negatively associated with Type Ⅱ interferon response (R = -0.38, *p* = 6.99×10^-14^, *n* = 361, **Figure 3B**). Immune cytolytic scoring revealed higher immune infiltration in the high-risk group but comparable stromal content in high- versus low-risk groups (**Figure 3C**).

To gain further resolution on the immunosuppressive microenvironment linked to high ALDH risk, we analyzed single-cell transcriptomics of 19,126 cells from 6 HCC patients 19. Unsupervised clustering defined 16 major cell populations, including M1 macrophages, M2 macrophages, CD4^+^ T cells, exhausted CD8^+^ T cells, Treg, NKT cells, natural killer (NK) cells, dendritic cells, B cells, tumor cells, AFP^+^ tumor cells, EPCAM^+^ tumor cells, PON^+^ tumor cells, MKI67^+^ progenitor cells, myeloid cells, and hepatic stellate cells (**Figure 3D**). Marker gene profiling highlighted the presence of exhausted CD8^+^ T cells, immunosuppressive M2 macrophages, and Tregs across patients (**Figure 3E**). Collectively, these integrated multi-omics analyses demonstrate an immunosuppressive microenvironment characterized by elevated Tregs infiltration, T cell dysfunction, and impaired anti-tumor immunity in the high ALDH subgroup.

### TNFRSF18 upregulation defines an immunosuppressive phenotype associated with high ALDH risk

**Figure 4:**
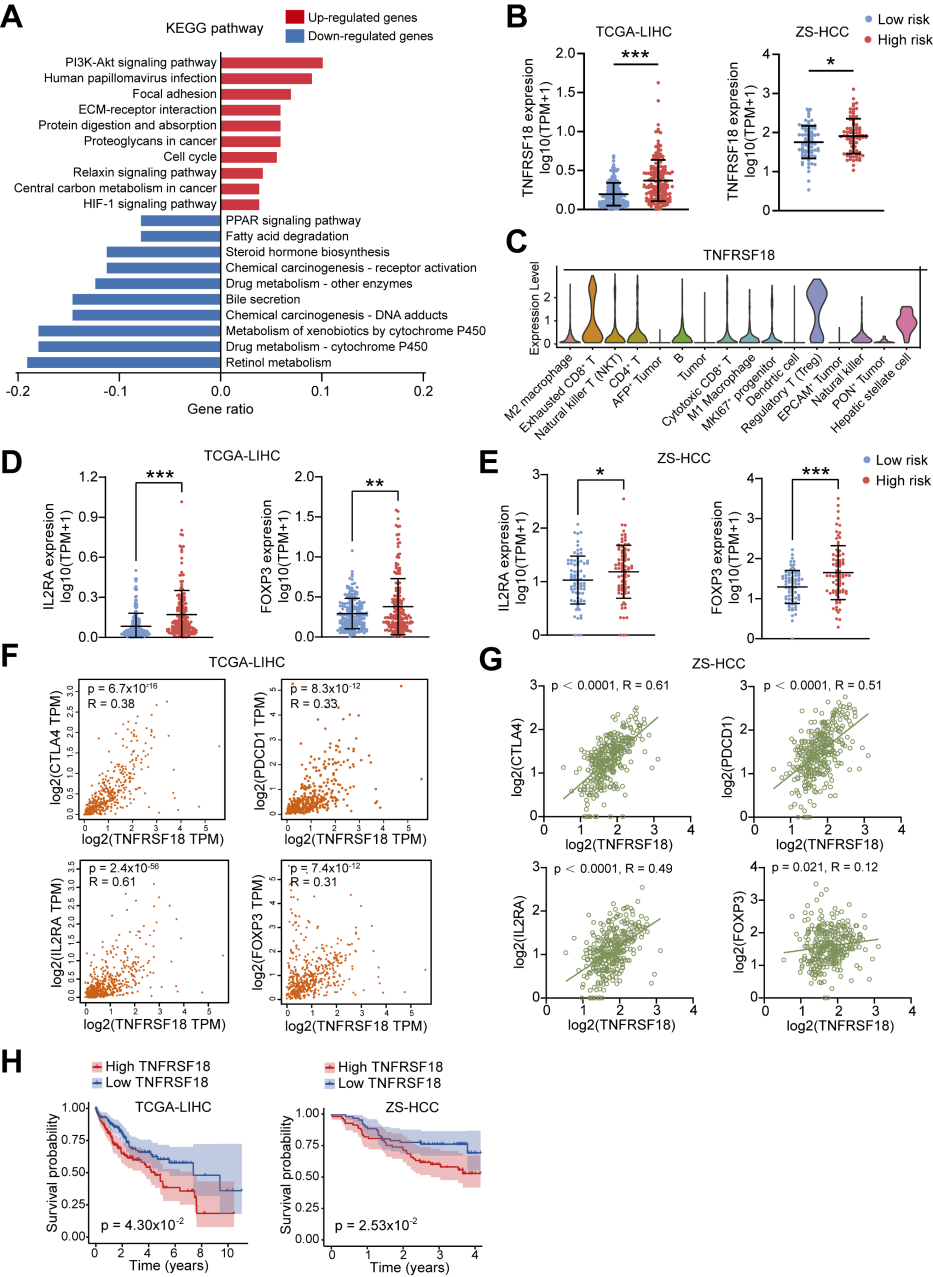
** TNFRSF18 upregulation defines an immunosuppressive, treatment-resistant phenotype in ALDHs high-risk HCC subset.** (A) Top 10 KEGG pathway enrichment of upregulated genes (red) in the high ALDH risk group showing associations with oncogenic signaling, while downregulated genes (blue) linked to metabolic processes. (B) TNFRSF18 exhibiting increased expression in the high versus low ALDH risk HCC patients across TCGA-LIHC and ZS-HCC cohorts. (C) Single-cell transcriptomics revealed selective TNFRSF18 expression within Treg cluster. (D, E) Classical Treg markers FOXP3 and IL2RA were upregulated in the high ALDH risk group in TCGA-LIHC (D) and ZS-HCC (E) cohorts. (F-G) TNFRSF18 expression positively correlated with immune checkpoint genes CTLA4, PDCD1 as well as Treg markers IL2RA, FOXP3 in TCGA-LIHC (F) and ZS-HCC (G) cohorts. (H) High TNFRSF18 expression stratified a subgroup with significantly reduced overall survival in TCGA-LIHC (left) and ZS-HCC (right) cohorts. Unpaired student's *t*-test was used in (B, D, E). Pearson correlation analysis was used in (F, G). Kaplan-Meier analysis was used in (H). * *p* < 0.05, ** *p* < 0.01, *** *p* < 0.001.

To dissect biological processes linked to the ALDH risk model, we compared differentially expressed genes between high- and low-risk HCC groups from TCGA-LIHC dataset. A total of 648 upregulated and 126 downregulated genes were identified in the high-risk subset (**Figure S3A**). KEGG pathway and GO enrichment analysis showed upregulated genes were enriched in oncogenic signaling such as PI3K-Akt, cell cycle, and HIF-1, while downregulated genes associated with metabolic processes including retinol, xenobiotic, and cytochrome P450 metabolism (**Figure 4A and Figure S3B-S3C**).

Intersecting the upregulated genes with a selected TME cell markers revealed 11 candidate drivers of the high-risk phenotype, including TNFRSF18 (**Figure S3D and Table S1**), a known marker of activated Tregs linked to immunosuppression 21, 22. Supporting this, high ALDH risk scores correlated with elevated TNFRSF18 mRNA expression across two HCC cohorts (**Figure 4B**). Single-cell transcriptomics confirmed selective TNFRSF18 expression within the Treg compartment (**Figure 4C**). Accordingly, classical Treg markers IL2RA and FOXP3 were upregulated in the high-risk HCC group across independent datasets (**Figure 4D-E**).

TNFRSF18 levels positively associated with expression of immune checkpoints CTLA4, PDCD1 as well as IL2RA and FOXP3 in both TCGA-LIHC (**Figure 4F**) and validation (**Figure 4G**) HCC cohorts. To be noted, high TNFRSF18 expression stratified a subgroup with significantly worse overall survival in TCGA-LIHC (*p* = 0.043) and ZS-HCC (*p* = 0.025) sets (**Figure 4H**). Collectively, these multi-omics analyses uncovered TNFRSF18 as a key upregulated target in the immunosuppressive, Tregs-enriched, and clinically aggressive high ALDH risk microenvironment, highlighting its potential as a therapeutic vulnerability.

### ALDH2 as a Metabolic Regulator of Tregs Infiltration in HCC

**Figure 5:**
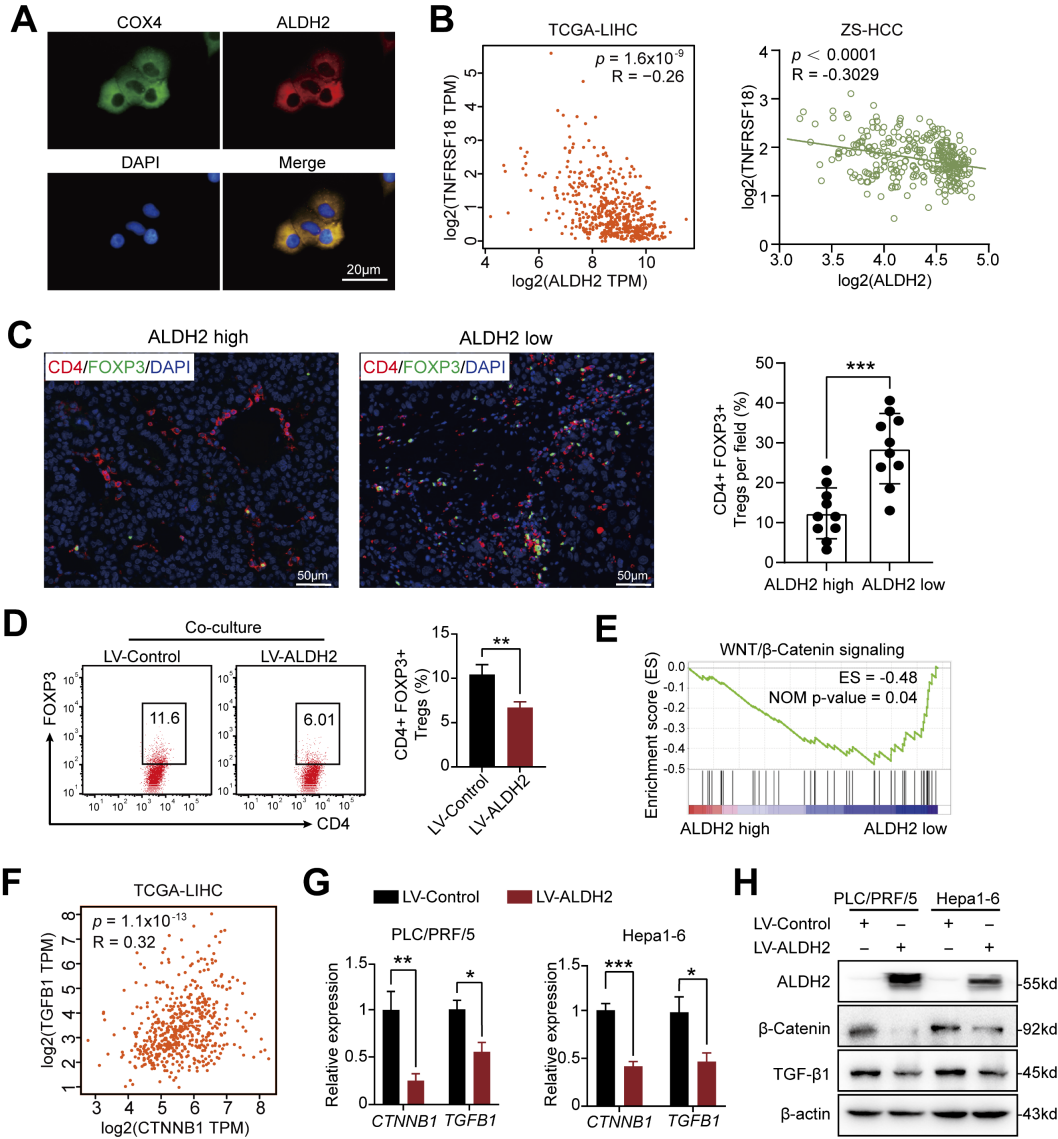
** ALDH2 overexpression suppresses Tregs differentiation in HCC via Inhibition of the β-Catenin/TGF-β1 Signaling.** (A) Immunofluorescence demonstrating mitochondrial localization of ALDH2 (red) in HCC cells, co-stained with mitochondrial marker COX4 (green). (B) ALDH2 mRNA levels inversely correlated with TNFRSF18 expression in TCGA-LIHC and ZS-HCC cohorts. (C) Immunofluorescence quantification of CD4^+^FOXP3^+^ Tregs infiltration in ALDH2-high and -low HCC specimens. (D) *In vitro* co-culture of Hepa1-6 cells overexpressing ALDH2 with CD4^+^ T cells revealed reduced differentiation of CD4^+^FOXP3^+^ Tregs. (E) Gene set enrichment analysis showed negative association between ALDH2 expression and the WNT/β-catenin signalling. (F) ALDH2 mRNA levels inversely correlated with CTNNB1 and TGFB1 expression in TCGA-LIHC cohort. (G-H) ALDH2 overexpression suppressed CTNNB1, TGFB1 mRNA (G) and protein (H) levels in HCC cells. Unpaired student's *t*-test was used in (C, D, G). Pearson correlation analysis was used in (B, F). * *p* < 0.05, ** *p* < 0.01, *** *p* < 0.001.

Among the prognostic ALDH genes identified, we prioritized ALDH2 for further mechanistic investigation based on its independent prognostic association with overall survival in multivariate analysis of the ZS-HCC cohort, alongside preoperative AFP levels (**Table 2**). Single-cell transcriptomics revealed selective ALDH2 expression within the tumor cell compartment (**Figure S4A and S4B**), which was downregulated in 30 pairs of HCC tissues compared to paired non-tumor tissues at mRNA and protein levels (**Figure S4C-E**). Immunofluorescence confirmed mitochondrial localization of ALDH2 (**Figure 5A**), consistent with its role in aldehyde detoxification 10.

Integrating ALDH2 expression with our previous TNFRSF18/Treg infiltration findings revealed a significant negative correlation between these two factors in TCGA-LIHC (TNFRSF18: R = -0.26, *p* = 1.6×10^-9^) and ZS-HCC (R = -0.30, *p* < 0.0001) cohorts (**Figure 5B**). Immunofluorescence quantification of CD4^+^FOXP3^+^ Tregs in 20 HCC specimens stratified by ALDH2 levels further supported an inverse relationship, with ALDH2-high tumors exhibiting markedly reduced Treg infiltration (**Figure 5C**). Together, these results suggest that ALDH2 downregulation promotes an immunosuppressive microenvironment for HCC progression, mediated in part through elevated Treg recruitment.

### ALDH2 inhibits Tregs infiltration *via* suppression of the β-Catenin/ TGF-β1 signaling

To delineate mechanisms by which ALDH2 regulates HCC pathogenesis, we engineered ALDH2 overexpression in human (PLC/PRF/5) and mouse (Hepa1-6) HCC cell lines (**Figure S4F**). ALDH2 overexpression potently inhibited cancer cell colony formation and proliferation *in vitro* (**Figure S4G and H**). We then co-cultured naïve CD4^+^ T cells isolated from mouse lymphoid organs (**Figure S4I**) with control or ALDH2-overexpressing Hepa1-6 cells. Notedly, ALDH2-overexpressing Hepa1-6 cells markedly impaired Treg differentiation in co-culture (**Figure 5D**).

**Figure 6:**
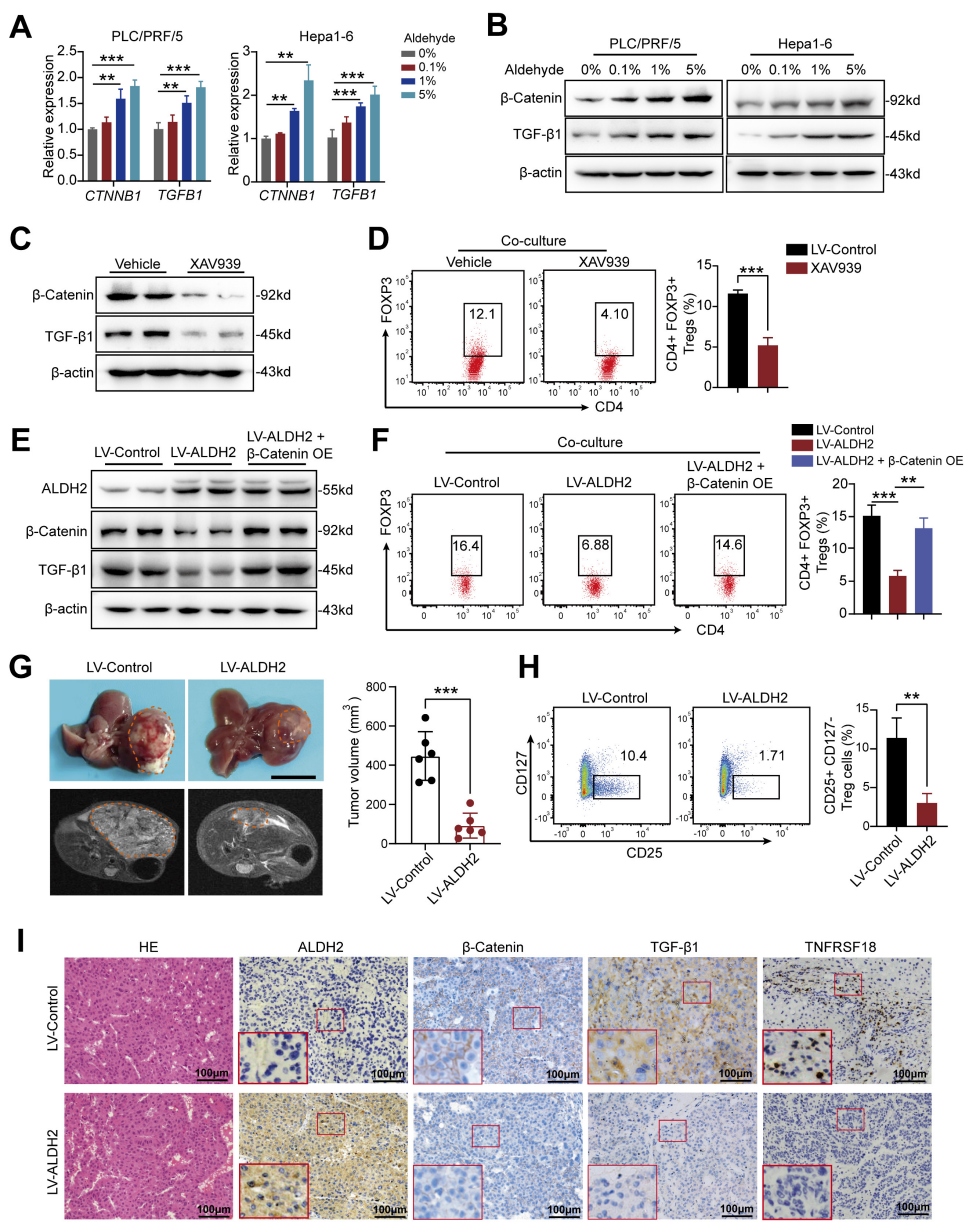
** ALDH2 overexpression inhibits HCC development *via* suppression of the β-Catenin/TGF-β signaling.** (A-B) Extrinsic aldehyde increased the mRNA (A) and protein levels (B) of CTNNB1 and TGFB1 in a concentration-dependent manner in HCC cells. (C) XAV939, a WNT/β-Catenin inhibitor, inhibited protein levels of β-Catenin and TGF-β1. (D) XAV939 treatment inhibited the differentiation of CD4^+^FOXP3^+^ Treg in a co-culture assay with Hepa1-6 cells. (E) Western blot analysis showing ALDH2 overexpression downregulated β-Catenin and TGF-β1, which was rescued by β-Catenin overexpression. (F) ALDH2 overexpression attenuated the differentiation of CD4^+^FOXP3^+^ Treg in a co-culture with CD4^+^ T cells, which was rescued by β-Catenin overexpression. (G) Bright-field and magnetic resonance imaging showing ALDH2 overexpression inhibited HCC development in orthotopic HCC model. Scale bar, 1cm. (H) Flow cytometry analysis revealed lower infiltration of CD4^+^CD25^+^CD127^-^ Treg in ALDH2-overexpressing HCC tumors. (I) Immunohistochemistry staining showed decreased protein levels of ALDH2, β-Catenin, TGF-β1, and TNFRSF18 in ALDH2-overexpressing HCC tumors. Unpaired student's t-test was used in (D, G, H). one-way ANOVA analysis was used in (A, F). * *p* < 0.05, ** *p* < 0.01, *** *p* < 0.001.

Gene set enrichment analysis (GSEA) of TCGA-LIHC dataset revealed an inverse correlation between ALDH2 expression and the WNT/β-catenin signaling (**Figure 5E**), a key oncogenic pathway that promotes tumor growth 23, 24. Accumulating evidence suggests that activated β-Catenin enhances TGF-β1 expression and promotes Treg biology 25-27. Consistent with these findings, our analysis revealed a positive correlation between β-Catenin (also known as CTNNB1) and TGF-β1 expression in TCGA-LIHC (*p* = 1.1×10^-13^, R = 0.32) and ZS-HCC (*p* = 0.0058, R = 0.1488) datasets (**Figure 5F and Figure S5A**). Ectopic ALDH2 overexpression in Hepa1-6 cells suppressed CTNNB1 and TGFB1 mRNA and protein levels (**Figure 5G and H**). Conversely, low ALDH2 activity can lead to aldehyde accumulation, and extrinsic aldehyde promoted reactive oxygen species (ROS) generation (**Figure S5B**) and concentration-dependent upregulation of TGF-β1 and β-Catenin in HCC cells (**Figure 6A and 6B**). Our findings suggested that ALDH2 inhibits an immunosuppressive microenvironment by constraining the signaling pathways of β-catenin and TGF-β1, which prime stemness programs linked to Treg differentiation.

To validate TGF-β1 as a downstream target of β-Catenin signaling, we increased β-Catenin expression, resulting in elevated TGF-β1 levels in HCC cells (**Figure S5C**). Besides, treatment with the β-Catenin inhibitor XAV939 downregulated β-Catenin and TGF-β1 protein levels in PLC/PRF/5 and Hepa1-6 cells (**Figure 6C**), and inhibited Treg differentiation (**Figure 6D**). Next, we upregulated β-Catenin in ALDH2-overexpressing HCC cells to examine its effect on colony formation and cancer proliferation. The results demonstrated that β-Catenin overexpression rescued TGF-β1 expression (**Figure 6E**) and partially restored colony formation and proliferation in HCC cells (**Figures S5D and E**). Moreover, β-Catenin overexpression rescued the attenuated Treg differentiation in ALDH2-overexpressing Hepa1-6 cells co-cultured with CD4^+^ T cells (**Figure 6F**).

To investigate the anti-tumor role of ALDH2 *in vivo*, we constructed an orthotopic HCC mouse model using Hepa1-6 cells expressing ALDH2 or a control vector. Bright-field and magnetic resonance imaging (MRI) showed that ALDH2 overexpression inhibited HCC development **(Figure 6G)**. Flow cytometry analysis revealed a lower proportion of CD4^+^CD25^+^CD127^-^ Treg in ALDH2-overexpressing HCC tissues compared to controls (**Figure 6H and Figure S5F**). Furthermore, IHC staining demonstrated attenuated protein expression of β-Catenin, TGF-β1, and TNFRSF18 in ALDH2-overexpressing HCC tissues (**Figure 6I**). Collectively, these results suggest that high ALDH2 levels inhibit Treg differentiation through suppression of the β-Catenin/TGF-β1 signaling, thereby repressing HCC development.

### ALDH2 protein phosphorylation is modified by PRKCZ and associated with the prognosis of HCC patients

**Figure 7:**
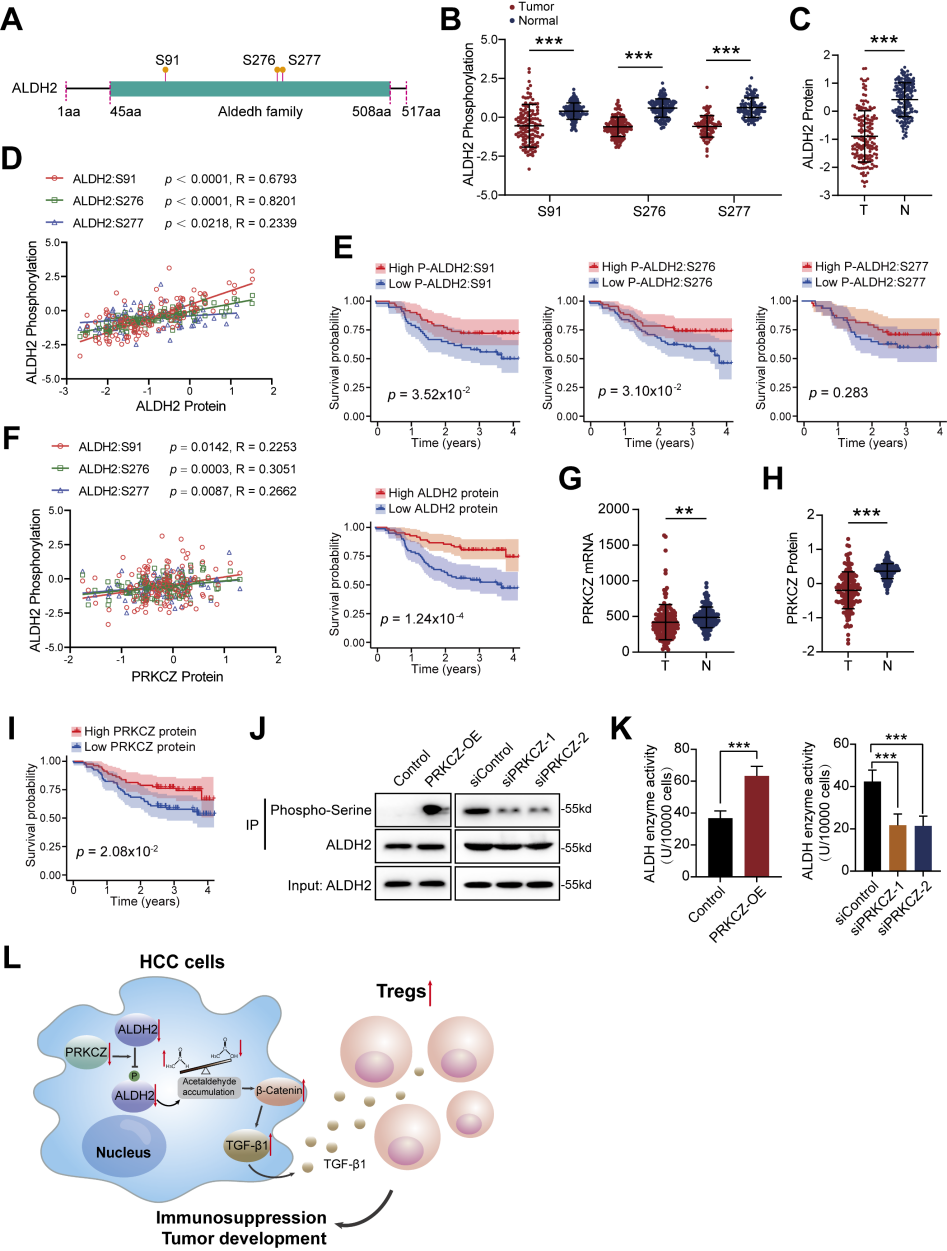
** PRKCZ mediates ALDH2 phosphorylation and is associated with prognosis in HCC.** (A) Three novel phosphorylation modification sites of ALDH2 (S91, S276, S277) were identified in HCC. (B) The phosphorylation levels of these ALDH2 sites were decreased in HCC tissues compared to normal liver tissues. (C) ALDH2 protein levels were down-regulated in HCC tissues. (D) ALDH2 protein levels positively correlated with its phosphorylation levels at S91, S276, and S277. (E) Low phosphorylation levels of ALDH2 at S91 and S276, along with low ALDH2 protein levels, were associated with poor prognosis in HCC patients. (F) PRKCZ protein levels positively correlated with ALDH2 phosphorylation at S91, S276, and S277. (G-H) PRKCZ mRNA (G) and protein (H) expression were downregulated in HCC tissues compared to normal liver. (I) Low PRKCZ protein levels were associated with poor prognosis in HCC patients. (J) Immunoprecipitation assay showing PRKCZ overexpression promoted, while PRKCZ knockdown attenuated, ALDH2 serine phosphorylation in HCC cells. (K) PRKCZ overexpression increased, while PRKCZ knockdown decreased, ALDH enzymatic activity. (L) Schematic diagram illustrating that ALDH2 downregulation promotes HCC tumorigenesis by enhancing Treg differentiation through the β-Catenin/TGF-β1 signaling. Unpaired student's t-test was used in (B, C, G, H, K). Pearson correlation analysis was used in (D, F). Kaplan-Meier analysis was used in (E, I). ** *p* < 0.01, *** *p* < 0.001.

The enzymic activity of ALDH2 is subject to post-translational modification, including phosphorylation 10, 28, 29. The phosphorylation modification data in the ZS-HCC dataset was analyzed, and 3 ALDH2 protein phosphorylation sites were identified, including serine (S)91, S276, and S277 (**Figure 7A**). The modification levels of these three sites were significantly decreased in HCC tumor tissues compared to normal liver tissues (**Figure 7B**). In total, 34 phosphorylation sites in ALDH family members were identified, most of which displayed attenuated phosphorylation levels in HCC tumors (**Figure S6A**). Meanwhile, ALDH2 protein expression was downregulated in HCC tumors (**Figure 7C**). ALDH2 protein levels were positively correlated with its phosphorylation levels at S91 (*p* < 0.0001, R = 0.68), S276 (*p* < 0.0001, R = 0.82), and S277 (*p* = 0.0218, R = 0.23; **Figure 7D**). This evidence suggests that ALDH2 and its phosphorylation levels are attenuated in HCC tumors, indicating low dehydrogenase activity in HCC patients. Kaplan-Meier analysis showed that low phosphorylation levels of ALDH2 at S91 (*p* = 3.52×10^-2^) and S276 (*p* = 3.10×10^-2^), along with low ALDH2 protein levels (*p* = 1.24×10^-4^), were associated with poor clinical outcomes (**Figure 7E**). However, no significant prognostic effect was observed for ALDH2 phosphorylation at S277 (*p* = 0.28; **Figure 7E**).

A previous study reported that PKCε modifies ALDH2 phosphorylation to prevent reperfusion arrhythmias in cardioprotection 30. In HCC patients, no significant correlation was found between ALDH2 phosphorylation and PKCε expression. However, another PRKC family member, PRKCZ, was identified as a mediator of ALDH2 phosphorylation (**Figure S7A**). PRKCZ protein levels positively correlated with ALDH2 phosphorylation at S91 (*p* = 0.0142, R = 0.23), S276 (*p* = 0.0003, R = 0.31), and S277 (*p* = 0.0087, R = 0.27; **Figure 7F**). PRKCZ mRNA and protein expression were downregulated in HCC tumors compared to normal liver tissues (**Figures 7G and 7H**). Survival analysis indicated that HCC patients with low PRKCZ protein expression had poor prognostic outcomes (**Figure 7I**).

Next, an IP assay was used to explore ALDH2 serine phosphorylation levels upon PRKCZ overexpression or knockdown in HCC cells (**Figures S7B and S7C**). Overexpressing PRKCZ promoted ALDH2 serine phosphorylation, while reducing PRKCZ attenuated ALDH2 serine phosphorylation (**Figure 7J**), consistent with the proteomic results. Moreover, ALDH enzyme activity increased in PRKCZ-overexpressing cells and decreased in PRKCZ-knockdown cells (**Figure 7K**). These results indicate that PRKCZ is a crucial mediator of ALDH2 enzyme activity through phosphorylation, further regulating Tregs infiltration in HCC progression (**Figure 7L**).

## Discussion

Accumulating evidence suggests that metabolic disturbances, such as dysregulated glucose metabolism, lipid homeostasis, bile acid metabolism, and steroid metabolism, contribute to progressive liver damage and HCC development 6, 31-33. Understanding the metabolic characteristics of HCC is crucial for revealing the pathophysiological mechanisms underlying this disease and identifying promising therapeutic strategies. Herein, we revealed the expression features of the ALDH family between HCC and normal tissues through multi-omics bioinformatic analyses and validated the anti-tumor role of ALDH2. PRKCZ can mediate the phosphorylation modification of the ALDH2 protein and regulate its enzymatic activity. High ALDH2 expression attenuated Treg infiltration via the β-Catenin/TGF-β1 pathway, thereby inhibiting HCC progression.

The ALDH family consisting of 19 members comprises a class of critical metabolic enzymes in the liver. Previous studies have shown that altered expression of ALDHs in HCC is associated with tumor development and chemoresistance 34, 35. For instance, ALDH6A1 participates in mitochondrial respiration, and its overexpression reduces nitric oxide levels but increases ROS levels in HCC cells 36. Additionally, ALDH18A1 exhibits aberrant expression in non-alcoholic steatohepatitis-associated HCC 37.

Another study demonstrates that inhibiting HDAC9 in HCC decreases ALDH1A3 expression, and the sphere formation ability of HCC cells is significantly suppressed by the ALDH inhibitor disulfiram 38. Moreover, ALDH2 overexpression alters acetaldehyde levels, reduces cellular redox status, activates the AMPK signaling pathway, and inhibits HCC development 39. In our study, we found that 15 out of 19 ALDH family members displayed transcriptional alterations in HCC tissues. Genetic mutations in ALDHs can lead to aberrant carbonyl metabolism and severe human diseases. A previous study identified 1350 common variants among the 19 ALDH members in the human genome 40. Although HCC patients generally exhibited low mutation rates in ALDH genes, those with ALDH alterations indicated poor prognostic outcomes. Using LASSO regression analysis, we identified four critical ALDH members for constructing a risk model that demonstrated satisfactory prognostic predictive efficiency for HCC patients in the training and validation cohorts. Moreover, HCC patients in the high-risk group showed immunosuppressive features.

The TME is a complex ecosystem, comprising malignant cells as well as various stromal and immune cell types 41. Understanding the dynamic interactions within the TME is crucial for developing effective cancer therapies. Through single-cell sequencing analysis, we investigated the TME features of HCC patients. Intriguingly, our data revealed that TNFRSF18 was primarily expressed by Tregs in the TME. Furthermore, TNFRSF18 expression was significantly upregulated in HCC patients with high ALDH activity, a marker of tumor-initiating cells. Importantly, high TNFRSF18 expression was associated with poor prognosis in HCC. We found that TNFRSF18 was primarily expressed by Tregs. Furthermore, TNFRSF18 was significantly upregulated in ALDH high-risk patients, and its high expression indicated poor prognosis for HCC patients. TNFRSF18 is a member of the TNFR superfamily that is widely expressed on Tregs and other activated immune cells. Previous studies have reported that TNFRSF18 expression is upregulated on Treg within the TME, and this expression is positively associated with their immunosuppressive function 42. Besides, TNFRSF18 has been identified as a marker of FOXP3^-^IL10^+^ Tr1 cells, a subtype of pTregs 43. Emerging evidence suggests that TNFRSF18-expressing Tregs represent a major immunosuppressive population of immune cells in many tumor types 44-46. Importantly, agonistic antibodies targeting TNFRSF18 have been shown to inhibit the expression of the coinhibitory receptor TIGIT (T cell immunoreceptor with immunoglobulin and tyrosine-based inhibitory motif (ITIM) domain) and deplete the suppressive function of Tregs 42, 47. In glioblastoma, a combination of anti-TNFRSF18 and anti-PD-1 antibodies exhibited satisfactory survival benefits by targeting Tregs, indicating this may be a promising strategy for anti-tumor immunotherapy 45.

ALDH2 displayed promising clinical predictive and prognostic value in our bioinformatic analyses. As an oxidoreductase, ALDH2 catalyzes the conversion of accumulated aldehydes from cellular metabolism and oxidative stress to reduce cytotoxicity and pathogenesis 10. The anti-tumor role of ALDH2 in HCC tumorigenesis has been previously reported 16, 39. In the present study, we identified that ALDH2 was negatively associated with TNFRSF18 expression in HCC. Moreover, our study revealed that upregulation of ALDH2 inhibited Treg differentiation by suppressing the β-Catenin/TGF-β1 signaling pathway in HCC development. ALDH2 enzyme activity can be regulated by post-translational modifications (PTMs), including phosphorylation and acetylation 10. While PKCε can phosphorylate and promote ALDH2 enzymatic activity in cardiac cells 30, we identified three novel phosphorylation sites in the ALDH2 protein mediated by the PRKCZ protein in HCC patients. Exogenous overexpression of PRKCZ increased ALDH2 phosphorylation and enzyme activity in HCC cells.

There are some limitations to our present study. Firstly, the ALDH family comprises 19 members with distinct enzyme features. Herein, we mainly investigated the expression differences and prognostic value of the ALDH family but did not focus on their enzyme functions in HCC. Besides, we identified ALDH2 as a critical tumor suppressor in HCC development, and its low expression was associated with the activation of the β-Catenin/TGF-β1 signaling pathways. Our study revealed that aldehyde accumulation in HCC caused by abnormal ALDH2 could increase the expression of CTNNB1 and TGF-β1. However, the exact molecular mechanism of β-Catenin/TGF-β1 signal activation mediated by ALDH2 needs further investigation.

In conclusion, our integrated multi-omics analysis identifies an ALDH-Treg-TNFRSF18 axis that contributes to HCC pathogenesis. Our study systematically explored the alterations of the ALDH family in HCC and revealed the correlations between aldehyde metabolic disturbance, immune cell infiltration, and tumorigenesis, which might provide therapeutic tools for clinical HCC treatment.

## Supplementary Material

Supplementary methods, figures and tables.

## Figures and Tables

**Table 1:** Univariate and multivariate Cox regression analyses of risk score from TCGA-LIHC cohort.

Variables	Univariate Cox		Multivariate Cox	
	HR (95%CI)	P value	HR (95%CI)	P value
Age (≥ 60 vs. < 60)	1.19 (0.84-1.70)	0.32	-	-
Gender (male vs. female)	0.81 (0.57-1.17)	0.27	-	-
Grade (G3 + 4 vs. G1 + 2)	1.13 (0.79-1.61)	0.50	-	-
Stage (III + IV vs. I + II)	2.50 (1.75-3.57)	4.65×10-7	2.26 (1.57-3.24)	1.04×10-5
Risk score (high vs. low)	2.78 (1.75-4.44)	1.72×10-5	2.38 (1.51-3.77)	2.03×10-4

**Table 2:** Univariate and multivariate Cox regression analyses of clinicopathological features and ALDH2, ALDH5A1, ALDH6A1, and ALDH8A1 expression from the ZS-HCC cohort.

Variables	Univariate Cox		Multivariate Cox	
	HR (95%CI)	P value	HR (95% CI)	P value
Age (≥ 60 vs. < 60)	0.67 (0.37-1.21)	0.19	-	-
Gender (male vs. female)	0.76 (0.41-1.41)	0.38	-	-
Liver cirrhosis (yes vs. no)	1.28 (0.70-2.35)	0.42	-	-
Tumor thrombus (yes vs. no)	2.19 (1.26-3.80)	5.34×10^-3^	0.73 (0.09-5.69)	0.77
Preoperative AFP level	1.00 (1.00-1.00)	4.00×10^-3^	1.00 (1.00-1.00)	0.047
Tumor number (> 1 vs. 1)	0.80 (0.43-1.48)	0.47	-	-
BCLC stage (C vs. A + B)	2.22 (1.29-3.84)	4.13×10^-3^	2.67 (0.29-24.81)	0.39
TNM stage (III + IV vs. I + II)	1.74 (1.02-2.95)	0.04	0.73 (0.25-2.11)	0.56
ALDH2 (high vs. low)	0.29 (0.16-0.52)	4.00×10^-5^	0.48 (0.23-0.98)	0.045
ALDH5A1 (high vs. low)	0.38 (0.22-0.67)	8.20×10^-4^	0.70 (0.36-1.34)	0.28
ALDH6A1 (high vs. low)	0.50 (0.29-0.86)	0.01	0.94 (0.50-1.80)	0.86
ALDH8A1 (high vs. low)	0.40 (0.23-0.69)	1.09×10^-3^	0.68 (0.35-1.33)	0.26
