# Supplementary material for: Multi-Omics profiling identifies aldehyde dehydrogenase 2 as a critical mediator in the crosstalk between Treg-mediated immunosuppression microenvironment and hepatocellular carcinoma
Source: Int J Biol Sci. 2024 May 5;20(7):2763–78. doi: 10.7150/ijbs.93075 (PMC11077362; doi:10.7150/ijbs.93075)
Supplement: Supplementary file 1 — Supplementary methods, figures and tables. [file ijbsv20p2763s1.zip › Supplementary information.pdf]

Supplementary material and methods

ALDH family expression and genetic variations analyses in HCC

The transcriptional expression level of the ALDH family in normal liver and HCC was explored using R's ggpubr and pheatmap packages. The expression correlation analysis of the ALDH family at the transcription level was examined using corrplot and psych packages in R. The mean log2 CNV values of each HCC patient were calculated and plotted in 23 chromosome pairs using the R Circos package.

Cox regression analysis and risk scores calculation

Univariate Cox regression analysis of the ALDH family was performed using the R survival package, and $p < 0.05$ was reserved. The candidates were further overlapped with differentially expressed ALDH family members to select the survival-related genes. The least absolute shrinkage and selection operator (Lasso) method was applied to obtain coefficients. The risk score formula was established by normalizing the expression of survival-related genes and weighing by Cox coefficients (risk score = -0.00633 × ALDH2 - 0.02161 × ALDH5A1 + 0.00779 × ALDH6A1 - 0.00613 × ALDH8A1). The timeROC package of R was utilized to plot the receiver operator characteristic (ROC) curve of risk scores in training and validation sets. The risk scores, survival-related genes, and clinical features of HCC patients were further entered into univariate Cox and multivariate Cox regression analysis using the R survival package.

Survival analysis and clinical prediction model construction

The survival and survminer packages of R were utilized for plotting Kaplan–Meier survival curves to show the survival status difference between the two groups. The log-rank test was used to evaluate the statistical significance. A clinical prediction model of the nomogram was constructed based on risk scores and clinical information of patients in training set from TCGA-LIHC and further validated in the ZS-HCC dataset. The calibration curve was generated by comparing the predicted value from the nomogram with the actual survival to assess the performance of the nomogram in training and validation sets.

Immune cell infiltration and immune score investigation

The infiltration scores of 16 types of immune cells and 13 kinds of immune functions in each HCC sample were calculated using single-sample gene set enrichment analysis (ssGSEA) in the R GSVA

package (21). The ESTIMATE package of R was performed to obtain the immune and stromal scores and quantified the immunological activity of each HCC patient using gene expression profiles (22). The relative abundance of tumor-infiltrating immune cells in high-risk and low-risk groups was compared using R ggpubr package. The correlation analysis between infiltration scores of immune cells and risk scores was explored using Pearson Correlation analysis and visualized using R ggstatsplot package.

Differentially expressed genes analyses between high-risk and low-risk groups

The differentially expressed genes between high-risk and low-risk were screened by R limma package with the criteria of FDR < 0.05 and $|\log2FC| > 1$. The heatmap of differentially expressed genes was plotted using Rpheatmap package. Gene ontology (GO) terms and Kyoto encyclopedia of genes and genomes (KEGG) pathways enrichment analyses were performed using R clusterProfiler package v 3.18.1.

Single cell data processing

The raw data of 6 HCC patients was downloaded from CNP0000650 dataset in CNGBdb for single-cell sequencing analysis. The Seurat package of R was utilized to process single-cell data, and the criteria were set as more than 4000, less than 11000 genes, and less than 3% mitochondria-related genes to filter low-quality cells. A total of 19126 cells were obtained for further analysis. The sequencing data were normalization, then the features of each sample were analyzed. Using the FindClusters of R package (resolution = 0.1), 8 types of cell populations were identified. UMAP was applied to reduce the dimension cell clustering. The cell populations were annotated using marker genes expressed in each cluster.

Proteome and phosphorproteome analyses

The proteome and phosphorproteome data were downloaded from ZS-HCC research in NODE database. Phosphorylation modification sites and protein expression levels of ALDH family members were retrieved for analysis. The prognostic value of protein phosphorylation in the ALDH family was explored using the R survival package. The correlation of phosphorylation modification and protein value was analyzed by R corrplot package and visualized using GraphPad Prism 9.

Culture of naïve $CD4^+$ T cells

Mouse naïve $CD4^+$ T cells were isolated from spleens and lymph nodes of 8-week-old C57BL/6N mice using a T cell isolation kit (Biolegend, California, USA). The purified $CD4^+$ T cells were

cultured in RPMI 1640 medium containing 10% FBS, 2 mg/mL anti-CD3 Ab (Biolegend, California, USA), 5mg/mL anti-CD28 Ab (Biolegend, California, USA), 5 ng/mL TGF-β1 (Biolegend, California, USA).

Plasmid construction, and transfection

The coding sequence (CDS) of PRKCZ and β-catenin were amplificated using PCR and subcloned into pEnCMV vector. ALDH2 was subcloned into pCDH-CMV lentiviral plasmid and was packaged using psPAX2 and pMD2G plasmids as previously described (6). The HCC cells were infected by indicated lentivirus supernatants with polybrene (5μg/mL). The siRNAs of PRKCZ were purchased from Tsingke Biotechnology (Shanghai, China). The HCC cells transfected with siRNAs and recombinant plasmids using Lipofectamine3000 (Invitrogen, California, USA). The sequences of siRNAs were list in supplementary table 2.

RNA isolation and real-time quantitative polymerase chain reaction (RT-qPCR)

Total RNA was isolated using Trizol reagent (Takara, Japan), then 1μg RNA were conducted to reverse transcription by Hifair® II 1st Strand cDNA Synthesis Kit (Yeasen, Shanghai, China). The qRT-PCR assay was performed using Hieff® qPCR SYBR Green Master Mix (Yeasen, Shanghai, China) following the manufacturer's instructions. The expression of β-actin was set as an internal control, and the relative expression levels of target genes were analyzed using the $2^{-\Delta\Delta CT}$ method. The PCR primers used in this study were listed in supplementary table 2.

Western blotting analysis

The clinical tissues and cells were lysed using RIPA buffer (NCM, Suzhou, China) with protease inhibitor cocktail (NCM, Suzhou, China), then centrifugated to obtain protein supernatants. The protein concentrations were determined using bicinchoninic acid (BCA) assay (WELLBI, Shanghai, China). The proteins were separated by SDS-PAGE and transferred onto PVDF membranes (Millipore, Bedford, USA). The bands were incubated with the primary antibodies anti-PRKCZ (Abclonal, 1:1000), anti-ALDH2 (Abclonal, 1:1000), anti-β-catenin (CST, 1:1000), anti-TGF-β1 (Abclonal, 1:1000), anti-β-actin (Abclonal, 1:1000) overnight at 4°C and followed with the corresponding secondary antibody incubation. The protein level of β-actin was applied as an internal control.

ALDH2 serine site phosphorylation and enzyme activity detection

The phosphorylation level of ALDH2 were measured by immunoprecipitation (IP). 3×10^6 indicated

HCC cells were harvested and incubated with ALDH2 primary antibody (Abclonal, 1:100) for 12 h at 4 °C. Then adding 40 μL Protein A magnetic beads (MedChemExpress, New Jersey, USA) into cell lysis and incubating for 2 h. The magnetic beads were washed 5 times using lysis buffer. Western-blotting was performed and incubated with pan Phospho-Serine antibody (Abclonal, 1:1000) to detect ALDH2 serine site phosphorylation level. The ALDH enzyme activity of indicated HCC cell extracts was analyzed by an ALDH activity assay kit (Sangon Biotech, Shanghai, China) according to the manufacturer's instructions.

Immunofluorescent (IF)

The HCC cells were plated on slides of a 24-well plate. After adherence, the cells were fixed with 4% paraformaldehyde, permeabilized by Triton X-100, and blocked with 5% bovine serum albumin. After that, the cells were incubated with primary antibodies: anti-ALDH2 (Abclonal, 1:200), anti-COX4 (Abclonal, 1:100) overnight at 4°C. After washing 3 times with PBST buffer, the cells were incubated with corresponding secondary antibodies. The nuclei of cells were stained with DAPI (WELLBI, Shanghai, China). Finally, the stained cells were visualized by a fluorescent microscope (Olympus, Tokyo, Japan).

Tissue IF analyses were performed in paraffin-embedded HCC sample slides. After deparaffinization and rehydration, the tissue slides were blocked by 5% bovine serum albumin (BSA), then incubated with primary antibodies: anti-CD4 (Abclonal, 1:200), anti-FOXP3 (Abclonal, 1:200) overnight at 4°C, followed by secondary antibodies conjugated with Alexa Fluor 488 and 594 (Invitrogen, 1:500). DAPI medium was applied for nuclear staining. Positive cells were detected by fluorescence microscope (Olympus, Tokyo, Japan) and quantified using ImageJ software.

Colony formation and CCK8 assays

For colony formation assay, 1,000 indicated HCC cells per well were planted 6-well plates and cultured for 14 days. The colonies were fixed with 4% paraformaldehyde and stained with crystal violet solution (Sangon Biotech, Shanghai, China). HCC cell growth was measured using the Cell Counting Kit-8 (CCK8) assay (Dojindo, Kumamoto, Japan) following the manual instruction. The CCK8 measurement was conducted every 12 hours at an absorbance of 450 nm. The cell growth curve was plotted using GraphPad Prism 9.

Coculture of HCC and T cells

Coculture experiment was performed in 24-well plates with an 8 μm transwell insert (Millipore,

Massachusetts, USA). The isolated $CD4^+$ T cells (3×10^5 cells) were planted in the upper chambers and indicated Hepa1-6 cells were seeded in the lower chambers for 48 h. $FOXP3^+$ Tregs were analyzed using flow cytometry.

Gene set enrichment analysis (GSEA)

The TCGA-LIHC data were divided into ALDH2 high- and ALDH2 low- groups according to the median expression value of ALDH2. GSEA v 4.1.0 were applied to analyze the expression data of two groups.

Immunohistochemistry (IHC)

For the immunohistochemistry (IHC) assay, the paraffin-embedded human and mouse HCC tissues were deparaffinized, rehydrated, and cut into tissue sections. The antibodies used for IHC staining were anti-ALDH2 (Abclonal, 1:200), anti-TNFRSF18 (CST, 1:400), anti-β-catenin (CST, 1:400), and anti-TGF-β1 (Abclonal, 1:400). IHC score was assessed based on the staining intensity and percentage of target protein.

Supplementary table 1. Differentially expressed marker genes of cell clusters in HCC.

Gene	Low Mean	High Mean	logFC	*p* value
UBE2C	7.515846	19.9915	1.411379	7.37E-13
TNFRSF18	0.673438	2.039185	1.598376	1.32E-12
CDK1	2.741774	5.984713	1.126173	5.79E-11
MDK	47.90378	111.7427	1.221969	2.47E-10
DKK1	2.720797	16.09173	2.564218	5.85E-08
CD3D	3.135768	7.572455	1.271942	1.26E-07
EPCAM	12.01045	31.53272	1.392559	2.86E-05
AFP	32.54158	394.7349	3.600528	5.87E-05
CD27	1.09821	2.413084	1.135724	0.000143
TFF3	3.362143	12.44733	1.888384	0.00018
PTGDS	31.35558	111.2073	1.826458	0.019458

Supplementary table 2. List of primers and siRNA sequences.

Gene name	Forward primer	Reverse primer
ALDH2 (human)	TTCCACAGGGGAGGTCATCT	ATAAGGCTTGCCGTTGTCCA
ALDH2 (mouse)	CTATACCCGCCATGAGCCTG	GGAAAGCCTGCCTCCTTGAT
PRKCZ (human)	ATCAAGTCCCACGCGTTCTT	ATCCTCATCGTCTGGGGTCA
CTNNB1 (human)	ATGACTCGAGCTCAGAGGGT	ATTGCACGTGTGGCAAGTTC
CTNNB1 (mouse)	ACTTGCCACACGTGCAATTC	ATGGTGCGTACAATGGCAGA
TGFB1 (human)	CGACTCGCCAGAGTGGTTAT	TAGTGAACCCGTTGATGTCCA
TGFB1 (mouse)	CTCCCGTGGCTTCTAGTGC	GCCTTAGTTTGGACAGGATCTG
β-actin (human)	CATGTACGTTGCTATCCAGGC	CTCCTTAATGTCACGCACGAT
β-actin (mouse)	GGCTGTATTCCCCTCCATCG	CCAGTTGGTAACAATGCCATGT

siRNA	Sequence
Control siRNA	GACAGCACAGAGAAGTCCACTATTT
PRKCZ siRNA 1	GGAGACAGATGGAATTGCTTACATT
PRKCZ siRNA 2	TGATGACGAGGATATTGACTGGGTA

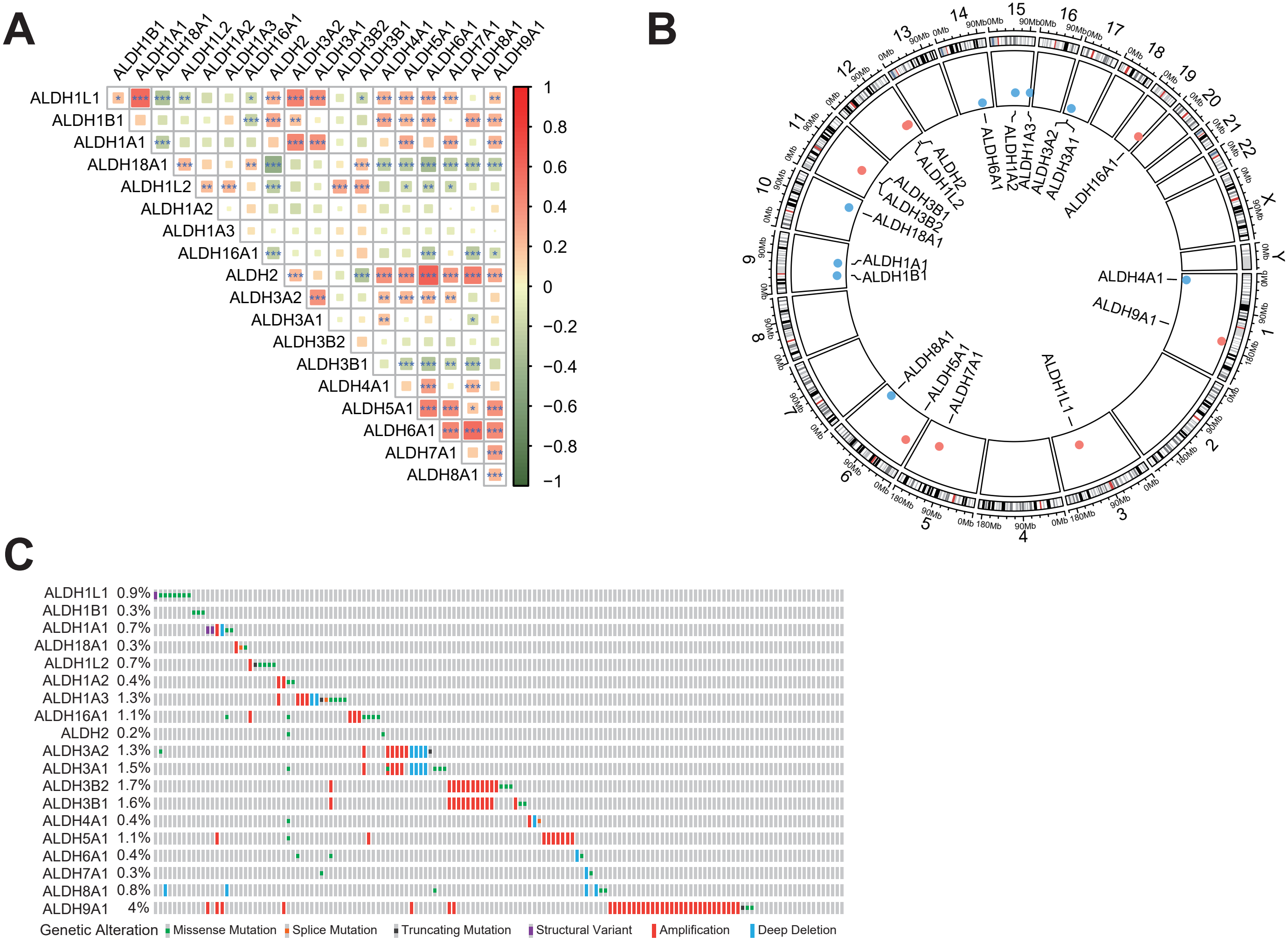

Figure S1. Genetic mutation analysis of the ALDHs in HCC. (A) Pearson correlation analyses between 19 ALDHs at the transcriptional level. Red and green dots represent positive and negative correlations, respectively. (B) The chromosome locations of CNV for 19 ALDHs. Red and blue dots indicate positive and negative values, respectively. (C) Genetic mutation rates of ALDHs in TCGA-LIHC dataset. Pearson correlation analysis was used in (A).

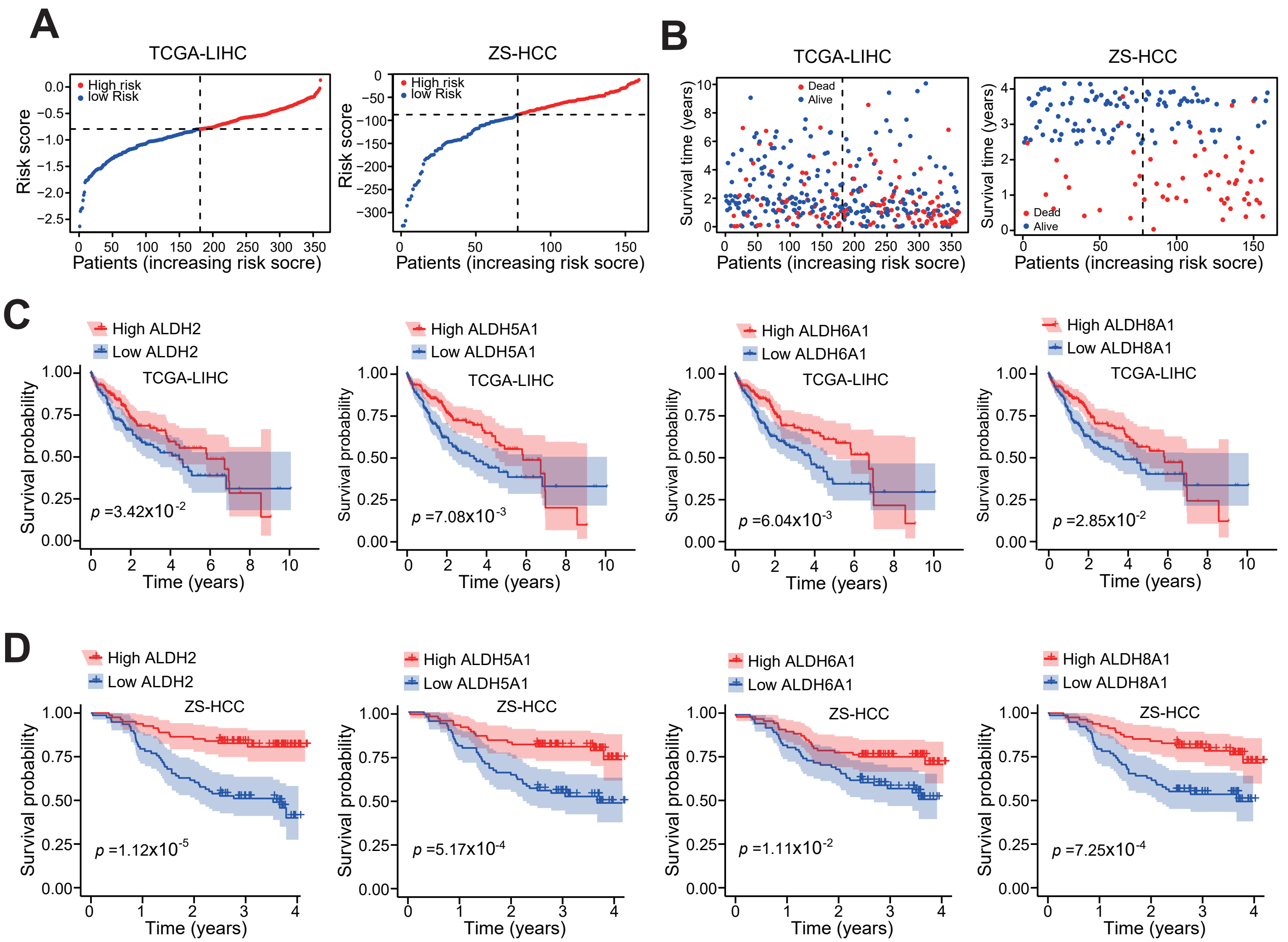

Figure S2. ALDHs risk scores analysis. (A) The HCC patients were divided into high-risk group and low-risk group based on the median risk score in TCGA-LIHC dataset and ZS-HCC dataset. (B) The dot plot showing the distribution of risk scores and survival times of patients from TCGA-LIHC and ZS-HCC dataset. (C) Kaplan–Meier analysis showing low expression of ALDH2, ALDH5A1, ALDH61, ALDH8A1 had poor overall survival probability in patients from TCGA-LIHC dataset. (D) Kaplan–Meier analysis howing low expression of ALDH2, ALDH5A1, ALDH61, andALDH8A1 had poor overall survival obability in patients from the ZS-HCC dataset.

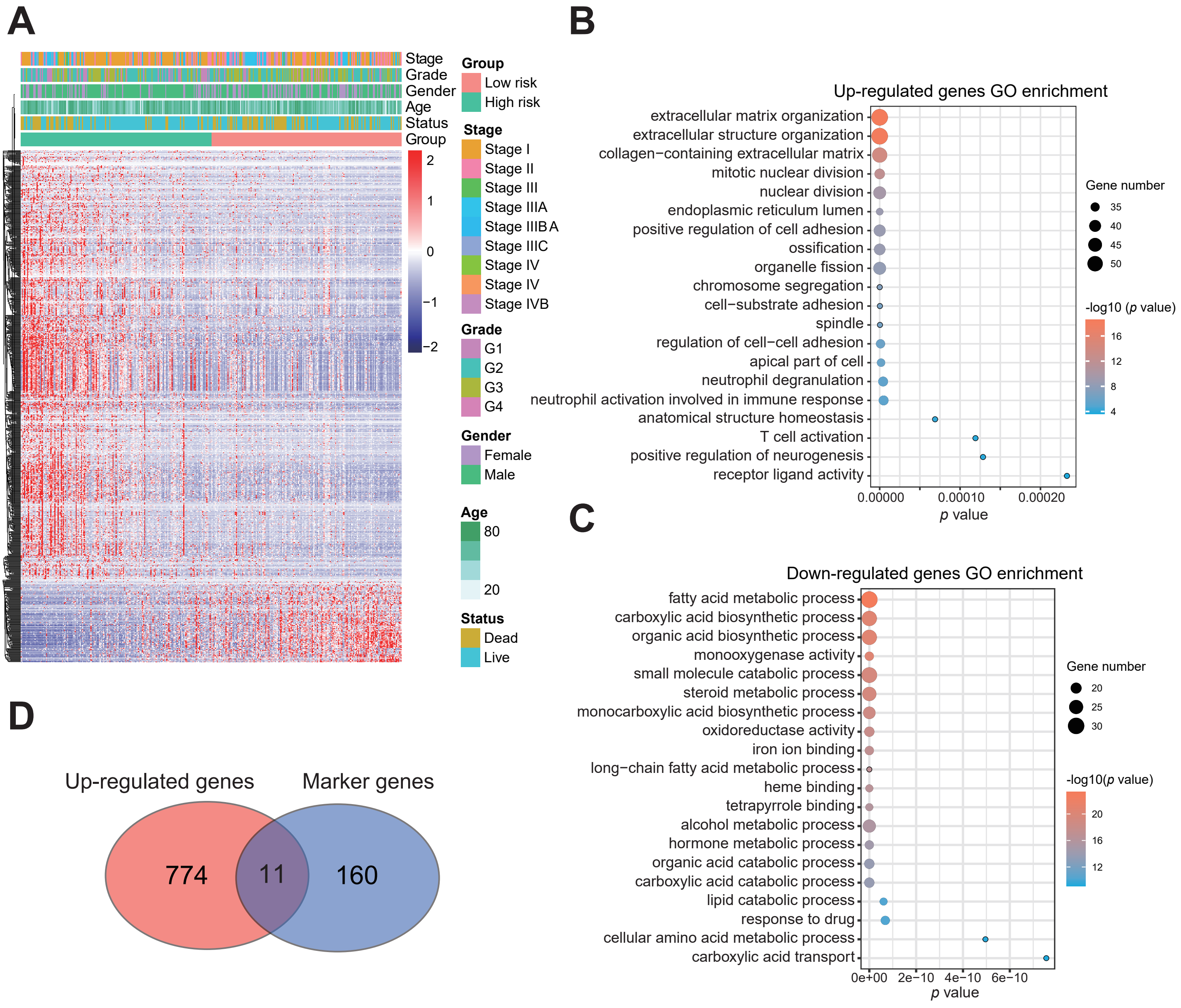

Figure S3. The differential gene expression and GO enrichment analysis between two ALDHs risk groups. (A) Heatmap showing the differentially expressed genes and clinical information between high and low ALDH risk groups. (B) GO enrichment analysis of up-regulated genes. (C) GO enrichment analysis of down-regulated genes. (D) Venn diagram of up-regulated genes and cell type markers in TME.

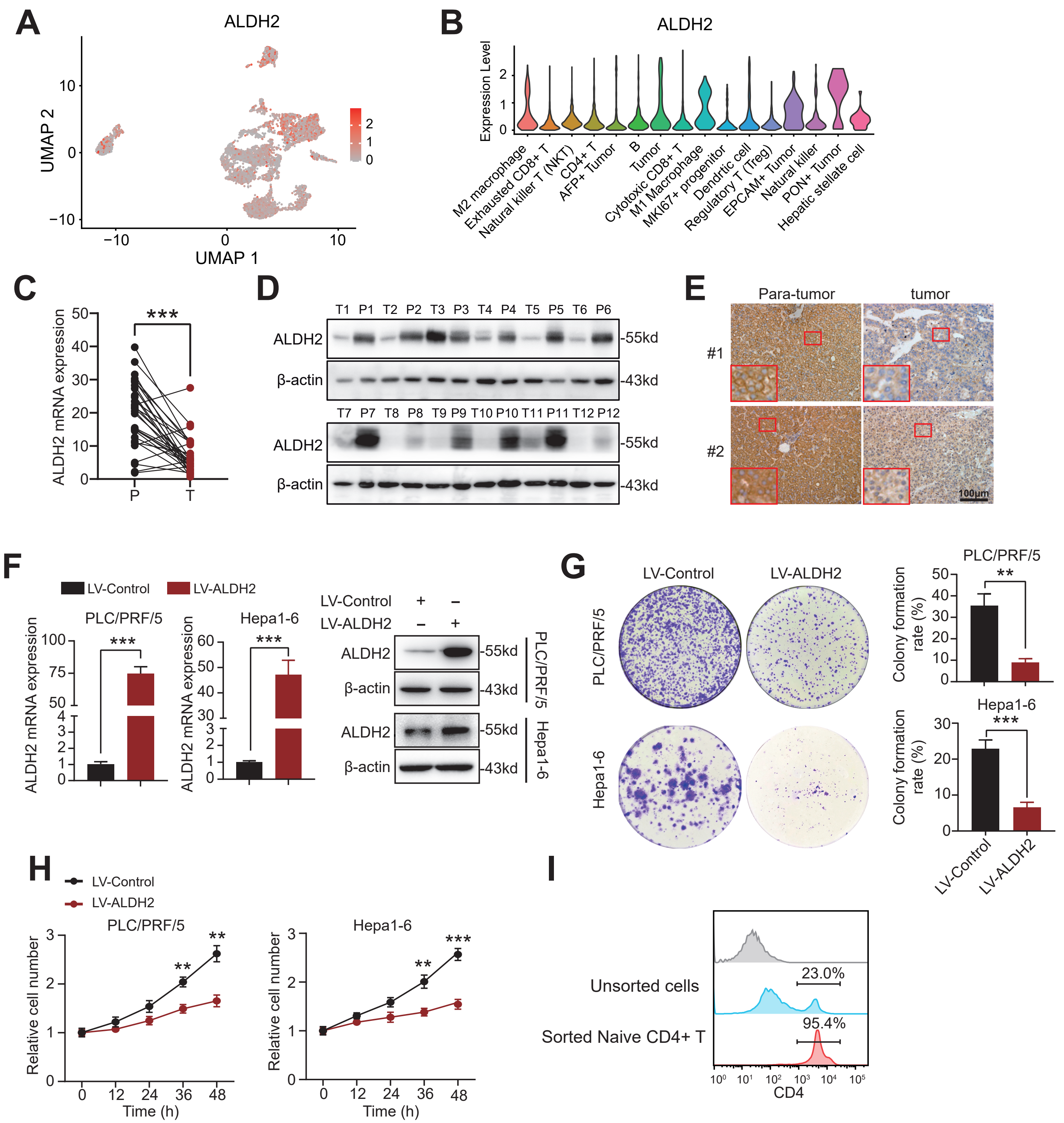

Figure S4. ALDH2 inhibits HCC development. (A) UMAP plot showing the expression features of ALDH2 in the HCC microenvironment. (B) Violin plot showing ALDH2 was widely expressed in tumor cells. (C) The mRNA level of ALDH2 was examined in para-tumor (P) and tumor (T) tissues from 30 HCC patients. The protein level of ALDH2 in clinical HCC tissues was explored by Western blot (D) and IHC (E). (F) ALDH2 overexpressing effects were determined by qPCR and Western blot in HCC cell lines. Clone formation (G) and CCK8 assays (H) were used to explore the proliferation of HCC cell lines between control and ALDH2 overexpressing groups. (I) Naive CD4+ T cell sorting efficiency was investigated by FCM. Unpaired student's t-test was used in (A, E, G, H). **p < 0.01, ***p < 0.001.

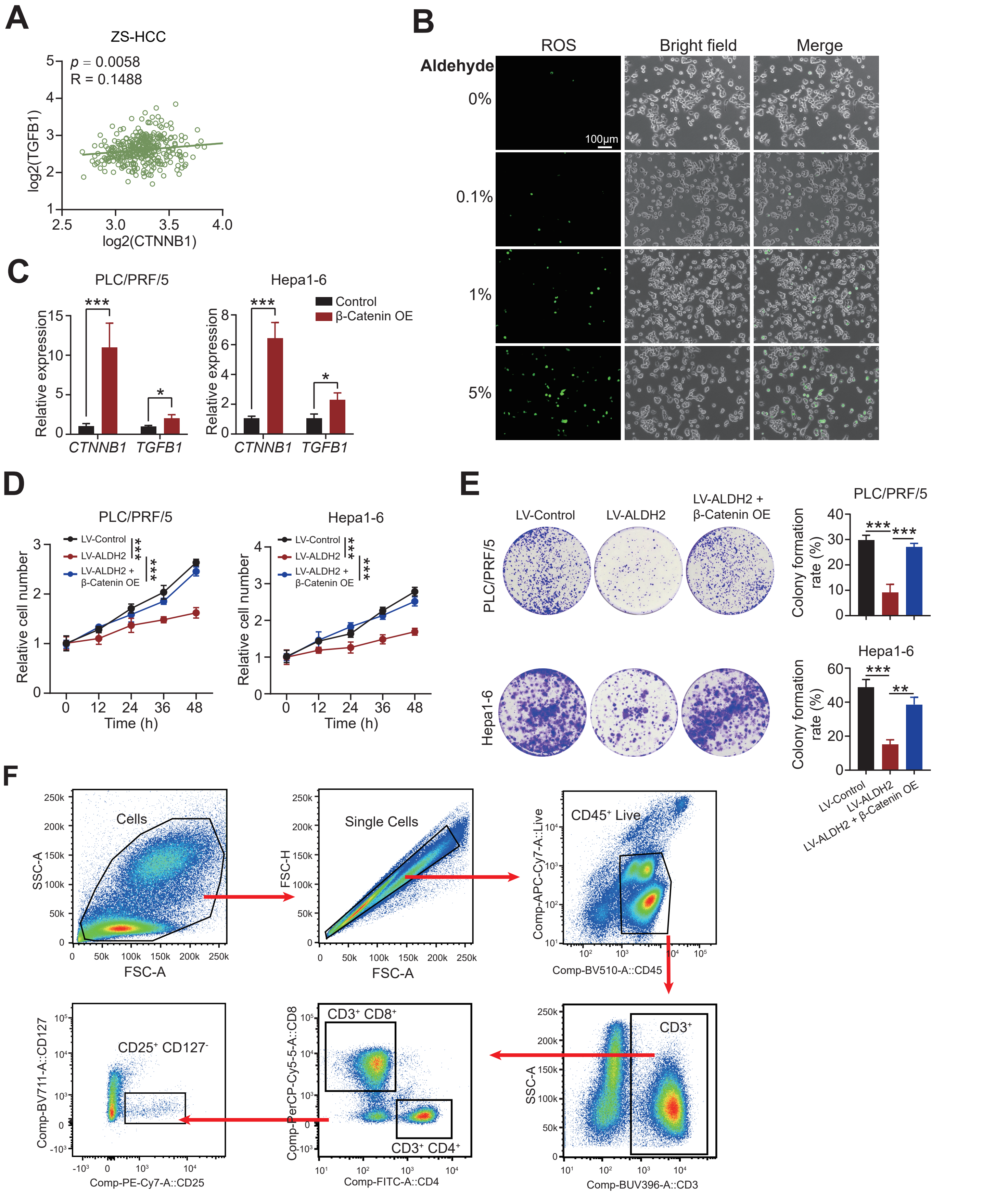

Figure S5. ALDH2 inhibits HCC development via β-Catenin/TGF-β1 signal. (A) Corrleration analysis between CTNNB1 and TGFB1 in ZS-HCC dataset.(B) Aldehyde promoted ROS generation in HCC cells. (C) CTNNB1 promoted TGFB1 expression in HCC cells. Clone formation (D) and CCK8 assays (E) were explored in ALDH2 overexpressing HCC cells with or without β-Catenin overexpression. (F) The analysis flow of flow cytometry.. Pearson corrleration analysis was used in (A). Unpaired student's t-test was used in (C). one-way ANOVA analysis was used in (D, E). **$p < 0.01$, ***$p < 0.001$, ****$p < 0.0001$, ns, not significant.

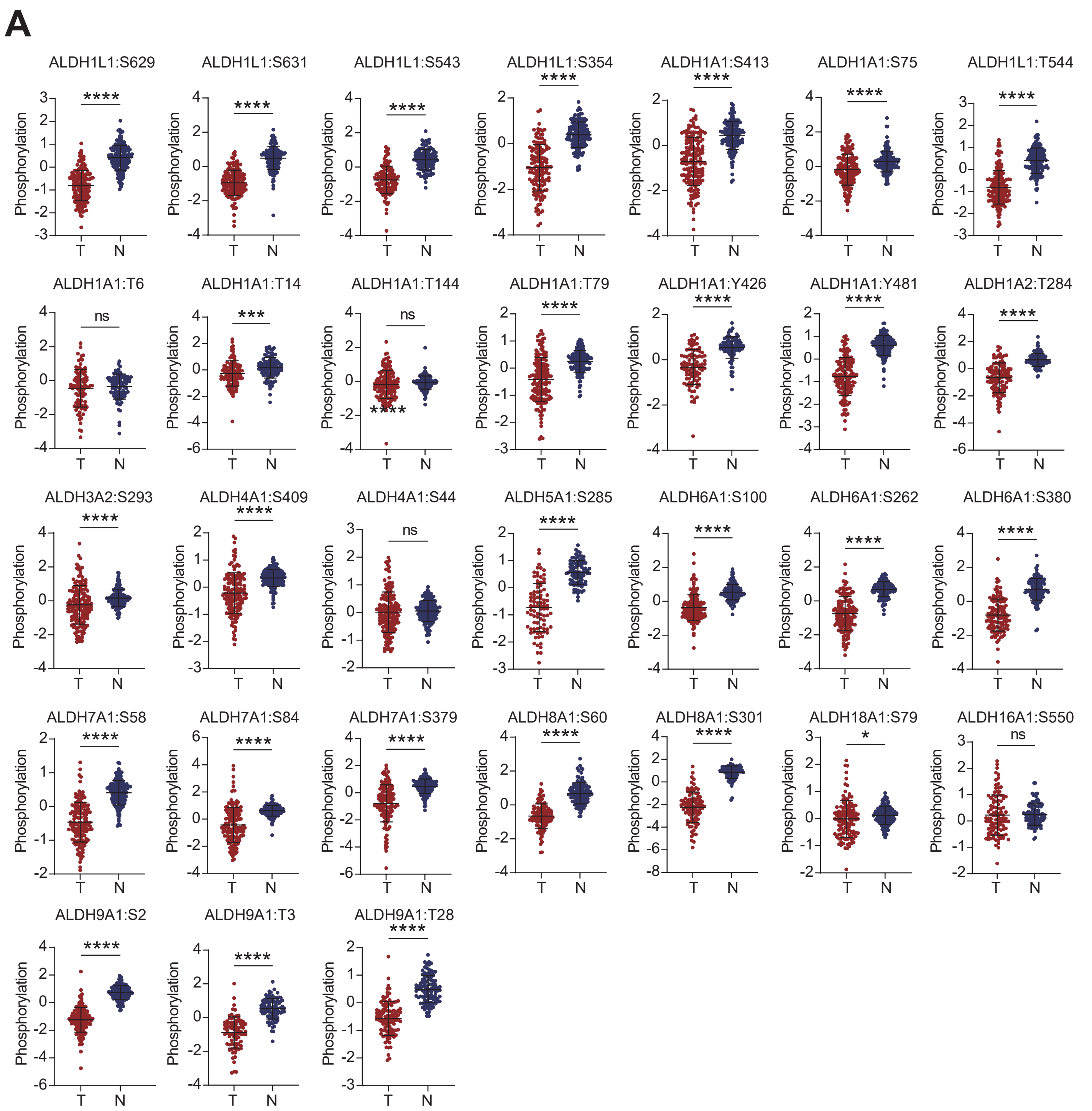

Figure S6A. Most of the phosphorylation levels of ALDHs were decreased in HCC tissues compared to normal liver tissues. Unpaired student's t-test was used in (A). **p < 0.01, ***p < 0.001, ****p < 0.0001, ns, not significant.

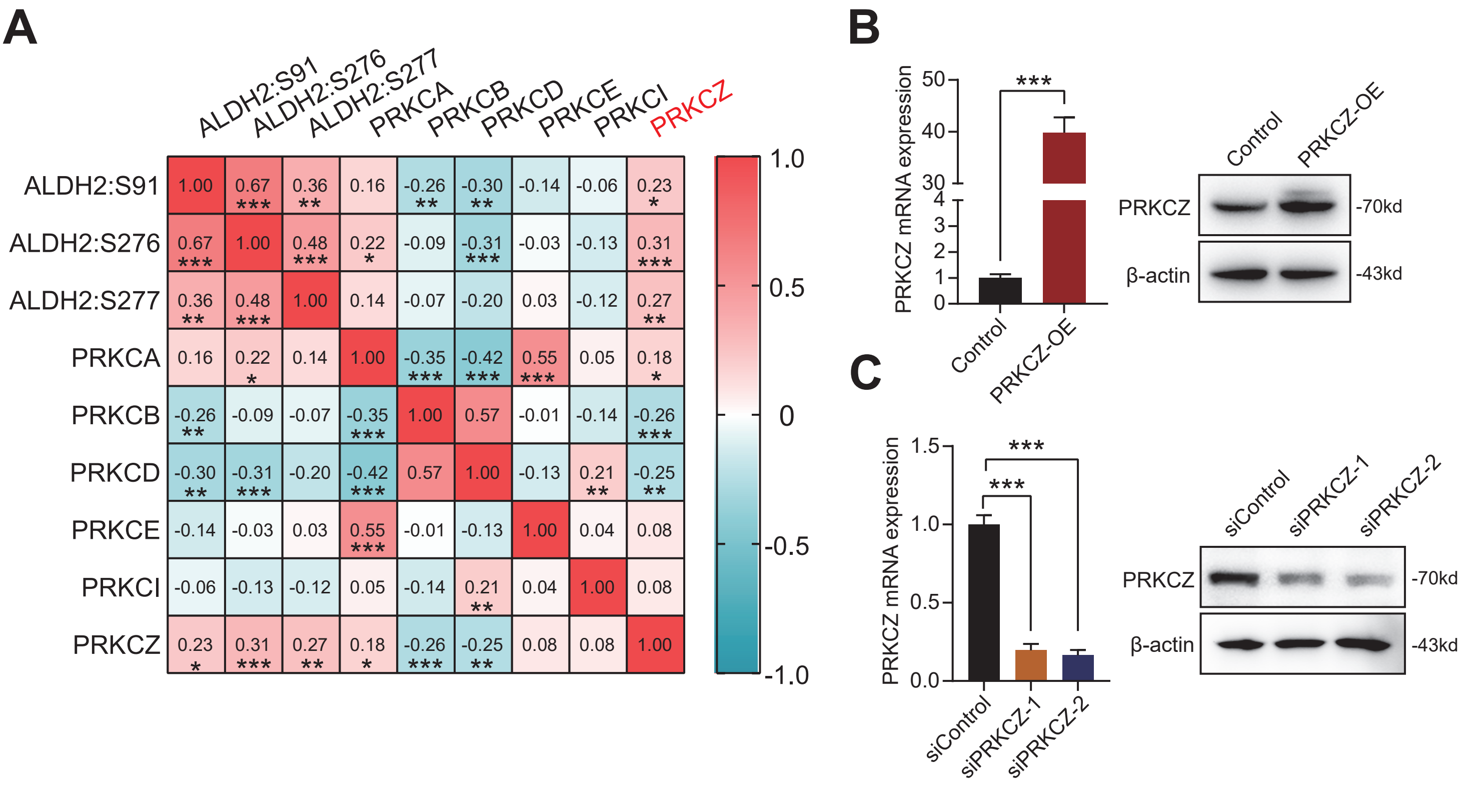

Figure S7. The phosphorylation of ALDH was correlated with PRKCZ expression in HCC. (A) The relationship between phosphorylation levels of ALDH2 and protein expressions of the PRKC family. PRKCZ overexpressing (B) and knockdown (C) effects were determined by qPCR and Western blot. Pearson correlation analysis was used in (A). Unpaired student's t-test was used in (B, C). * $p < 0.05$, ** $p < 0.01$, *** $p < 0.001$.
